# Fucosidases from the human gut symbiont *Ruminococcus gnavus*

**DOI:** 10.1007/s00018-020-03514-x

**Published:** 2020-04-24

**Authors:** Haiyang Wu, Osmond Rebello, Emmanuelle H. Crost, C. David Owen, Samuel Walpole, Chloe Bennati-Granier, Didier Ndeh, Serena Monaco, Thomas Hicks, Anna Colvile, Paulina A. Urbanowicz, Martin A. Walsh, Jesus Angulo, Daniel I. R. Spencer, Nathalie Juge

**Affiliations:** 1 The Gut Microbes and Health Institute Strategic Programme, Quadram Institute Bioscience, Norwich Research Park, Norwich, NR4 7UQ UK; 2 Ludger Ltd, Culham Science Centre, Abingdon, OX14 3EB UK; 3 Diamond Light Source Ltd, Diamond House, Harwell Science and Innovation Campus, Didcot, OX11 0DE UK; 4 Research Complex at Harwell, Harwell Science and Innovation Campus, Didcot, OX11 0FA UK; 5 School of Pharmacy, University of East Anglia, Norwich Research Park, Norwich, NR4 7TJ UK; 6 Present Address: The John Innes Centre, Norwich Research Park, Norwich, NR4 7UH UK; 7 Present Address: Departamento de Química Orgánica, Universidad de Sevilla, C/ Prof. García González, 1, 41012 Sevilla, Spain; 8 Present Address: Instituto de Investigaciones Químicas (CSIC-US), Avda. Américo Vespucio, 49, 41092 Sevilla, Spain

**Keywords:** Gut microbiota, Glycoside hydrolase, Mucus, Mucin glycosylation, Lewis epitopes, Antennary fucose

## Abstract

The availability and repartition of fucosylated glycans within the gastrointestinal tract contributes to the adaptation of gut bacteria species to ecological niches. To access this source of nutrients, gut bacteria encode α-l-fucosidases (fucosidases) which catalyze the hydrolysis of terminal α-l-fucosidic linkages. We determined the substrate and linkage specificities of fucosidases from the human gut symbiont *Ruminococcus gnavus*. Sequence similarity network identified strain-specific fucosidases in *R. gnavus* ATCC 29149 and E1 strains that were further validated enzymatically against a range of defined oligosaccharides and glycoconjugates. Using a combination of glycan microarrays, mass spectrometry, isothermal titration calorimetry, crystallographic and saturation transfer difference NMR approaches, we identified a fucosidase with the capacity to recognize sialic acid-terminated fucosylated glycans (sialyl Lewis X/A epitopes) and hydrolyze α1–3/4 fucosyl linkages in these substrates without the need to remove sialic acid. Molecular dynamics simulation and docking showed that 3′-Sialyl Lewis X (sLeX) could be accommodated within the binding site of the enzyme. This specificity may contribute to the adaptation of *R. gnavus* strains to the infant and adult gut and has potential applications in diagnostic glycomic assays for diabetes and certain cancers.

**Electronic supplementary material:**

The online version of this article (10.1007/s00018-020-03514-x) contains supplementary material, which is available to authorized users.

## Introduction

The microbial community inhabiting the human gut (gut microbiota) exerts a profound effect on human health through, e.g. polysaccharide digestion, metabolite and vitamin production, maturation of the immune system and protection against pathogens [1]. The adult gut microbiota is dominated by members of Firmicutes and Bacteroidetes phyla whereas the infant gut microbiota is dominated by Bifidobacterium that are adapted to utilize human milk oligosaccharides (HMOs), which are one of the major glycans found in breast milk. HMOs are composed of a linear or branched backbone containing galactose (Gal), *N*-acetylglucosamine (GlcNAc) and glucose (Glc), which can be decorated with fucose (Fuc) and/or sialic acid (Sia) residues, depending on the mother’s secretory status [2, 3]. In the adult colon, gut bacteria have not only access to non-digestible polysaccharides from the diet, but also to complex oligosaccharides from host mucins [4–6]. Mucins are large glycoproteins with a high carbohydrate content of up to 80%. Mucin-type *O*-glycans consists of *N*-acetylgalactosamine (GalNAc), Gal and GlcNAc, containing glycan chains modified by fucosylation, sialylation and sulfation [7–9]. The main source of glycan diversity is provided by the peripheral terminal epitopes that show considerable variation. The H1 structure (α1,2-fucose) is found in populations carrying the secretor gene [10], and individuals may also express the Lewis gene and the Lewis B (LeB) histoblood group antigen if they are secretors, while non-secretors express Lewis A (LeA) [11]. Another phenotype (SeW—weak secretor) is characterized by the expression of both LeA and LeB antigens [12]. The presentation of the major mucin glycan epitopes, sialic acid and fucose, varies along the GI tract with a decreasing gradient of fucose and ABH blood group expression and an increasing gradient of sialic acid from the ileum to the colon [7]. These gradients are reversed in mice, where the small intestine is dominated by sialylated structures and the colon with those terminating in fucose [13]. These glycans provide a potential source of nutrients to members of the gut microbiota [5]. In particular, α-l-fucosidases (α-fucosidases) are key enzymes for the degradation and metabolism of intestinal mucin glycans or HMOs by gut microbes and therefore, contribute to shaping the composition of the gut microbiota by favoring different bacterial species and influencing health and disease. Currently, α-fucosidases which catalyze the release of α-1–2, α-1–3, α-1–4 and α-1–6 linked fucose are classified into glycoside hydrolase (GH) families 29 and 95 (CAZy, www.cazy.org). All GH95 enzymes functionally characterized so far show strict substrate specificity to the terminal Fuc α1-2Gal linkage and hydrolyze the linkage via an inverting mechanism whereas GH29 enzymes show relatively relaxed substrate specificities with hydrolysis proceeding via a retaining mechanism (www.cazy.org). It was suggested that GH29 can be divided into two subfamilies. One contains fucosidases with relaxed substrate specificities that can act on 4-nitrophenyl α-l-fucopyranoside (*p*NP-Fuc) (referred to as GH29-A) (EC 3.2.1.51), whereas the members of the other subfamily show strict specificity for terminal α-(1–3/4)-fucosidic linkages with little/no activity on *p*NP-Fuc (GH29-B) (EC 3.2.1.111) as shown for fucosidases from *Streptomyces* and *Bacteroidetes thetaiotaomicron* [14, 15]. The GH29-A subfamily includes fucosidases from *Thermotoga maritima* [16], soil metagenome [17] or bacterial pathogens [18, 19] whereas the GH29-B subfamily includes fucosidases from *Bifidobacterium bifidum* (*Bb*AfcB) [20] *Clostridium perfringens* (*Cp*Afc2) [21] and *Streptococcus pneumoniae* (*Sp*GH29^c^) [22]. Despite the importance of fucose in regulating bacterial intestinal colonization in adults and infants, only a limited number of fucosidases have been studied at a biochemical level from human gut symbionts.

*Ruminococcus gnavus* is a prevalent member of the gut microbial community belonging to the Firmicutes division [23, 24]*. R. gnavus* is an early colonizer of the human gut [25] but persists in healthy adults where it belongs to the 57 species detected in more than 90% of human faecal samples by metagenomic sequencing [23]. In the past few years, an increasing number of studies are reporting a disproportionate representation of *R. gnavus* in diseases, such as inflammatory bowel disease [26]. In our previous work, we showed that *R. gnavus* ability to grow on HMOs or mucins was strain dependent [27, 28], underscoring the importance of analysing glycan utilization by members of the human gut microbiota at the strain level. These differences are reflected by the distribution of GH families between *R. gnavus* strains [27]. For example, *R. gnavus* E1 genome lacks a sialidase encoding gene whereas *R. gnavus* ATCC 29149 encodes a GH33 enzyme which has been functionally characterized as an intramolecular *trans*-sialidase [29] and is associated with a unique sialic acid metabolism pathway which forms the basis of *R. gnavus* ATCC 29149 adaptation to mucus [30]. In contrast, both *R. gnavus* E1 and *R. gnavus* ATCC 29149 genomes harbor fucosidase encoding genes belonging to GH29 or GH95 families [27], but their functional characterization has not been reported. To gain further biochemical and structural insights into *R. gnavus* strategy to utilize mucin glycans, we determined the substrate and linkage specificities of a range of fucosidases belonging to GH29 and 95 family from *R. gnavus* ATTC 29149 and E1 strains. We identified and characterized a fucosidase from *R. gnavus* E1 with the capacity to recognize fucosylated glycans capped with sialic acid and to hydrolyze α1–3/4 fucosyl linkages in these substrates without the need to remove sialic acid. This unique specificity may contribute to the adaptation of *R. gnavus* strains to distinct nutritional niches. Since changes in abundances of sialyl fucosylated epitopes on human glycans have been associated in several diseases, such as diabetes and certain cancers, these novel fucosidases may have potential in diagnostic glycomic assays.

## Materials and methods

### Materials

**Fig. 1:**
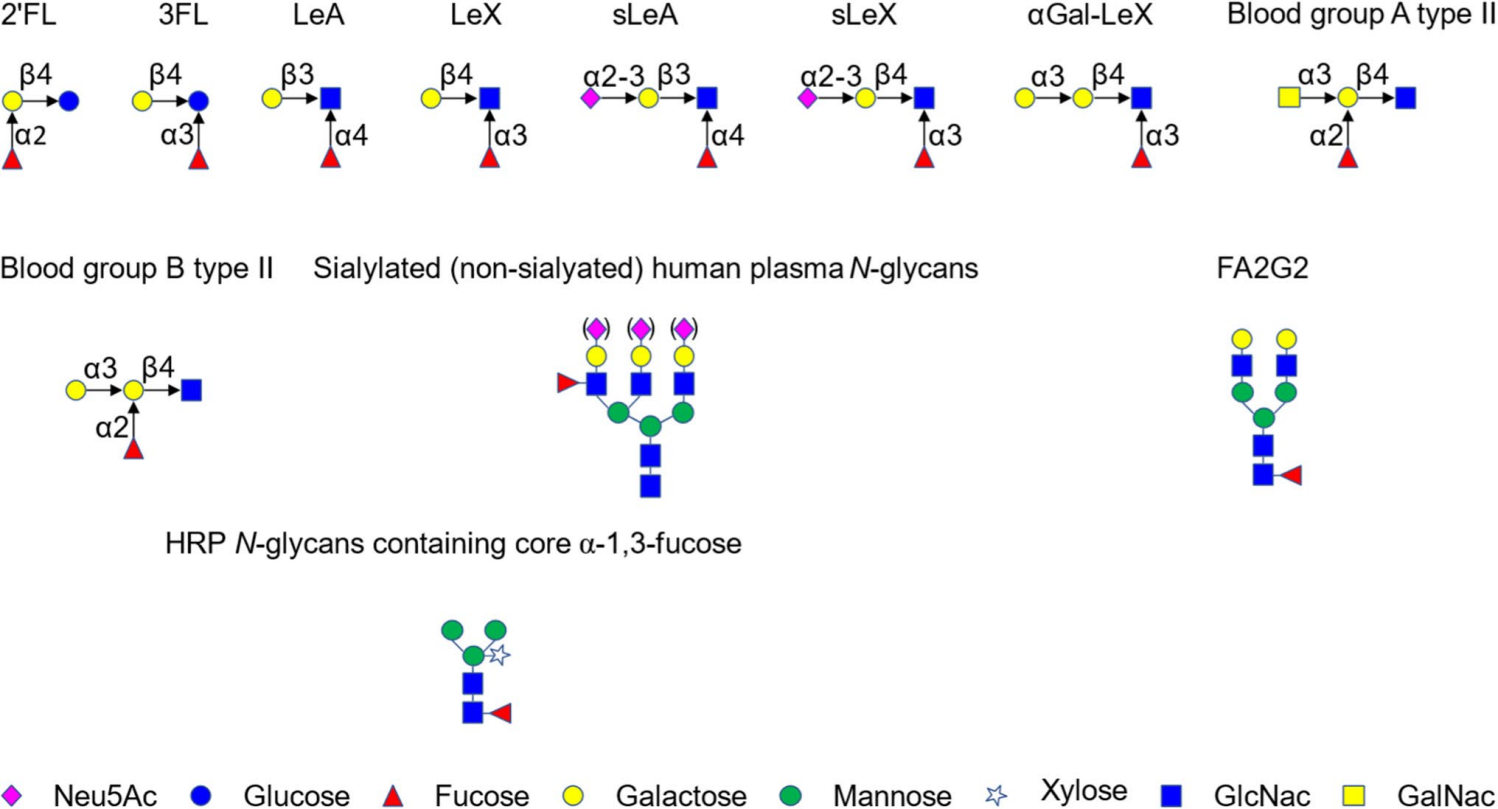
Fucosylated oligosaccharides used in this study. Monosaccharide symbols follow the Symbol Nomenclature for Glycans system [98]

All chemicals were obtained from Sigma (St Louis, MO, USA) unless otherwise stated. The structure of the oligosaccharides used in this work is shown in Fig. 1. 3′-Sialyl Lewis X (sLeX) was purchased from Carbosynth Limited (Campton, UK), Lewis A (LeA), α1–3Gal-Lewis X (αGal-LeX), Blood group A/B tetrasaccharide type II (Blood group A/B type II) were from Elicityl (Crolles, France), Lewis X (LeX) used for activity assay was from Dextra Laboratories (Reading, UK), LeX/LeA/*N*-Acetylneuraminic acid (Neu5Ac) used for ITC were from Carbosynth Limited (Campton, UK), LeX used for STD NMR was from the Consortium for Functional Glycomics (CFG). 3′-Sialyl Lewis A (sLeA), sialylated and desialylated human plasma *N*-glycans and FA2G2 *N*-glycans were from Ludger (Oxford, UK). Horseradish peroxidase (HRP) treated with BM03341 plant specific PNGase was a kind gift from Dr Lucy Crouch (Newcastle University). *E. coli* strain (Tuner DE3 pLacI) was from Merck (Darmstadt, Germany).

### Cloning, expression, mutagenesis and purification of GH29 and GH95 fucosidases

*Ruminococcus gnavus* ATCC 29149 or E1 genomic DNA (gDNA) was purified from the cell pellet of a bacterial overnight culture (1 ml) following centrifugation (5000*g*, 5 min) using the GeneJET Genomic DNA Purification Kit (ThermoFisher, UK), according to the manufacturer’s instructions. The full-length sequence of E1_10125 and E1_10180*,* excluding the signal sequence were cloned into the pOPINF expression system [31], introducing an His-tag at the N terminus. The D221A mutant of E1_10125 was produced by NZYTech (Lisbon, Portugal). The E1_10125G260M mutant was generated using the NZY Mutagenesis kit (Lisbon, Portugal). The ATCC_03833 and ATCC_00842 sequences exempt of the signal sequence were cloned into pET28a with N terminal His tag by Prozomix (Haltwhistle, UK). The E1_10587 was synthesized by NZYTech (Lisbon, Portugal) into pHTP1 with N terminal His tag.

The primers used are listed in Table S1. DNA manipulation was carried out in *E. coli* DH5α cells. Sequences were verified by DNA sequencing at Eurofins MWG (Ebersberg, Germany) or Earlham Institute (formerly TGAC, Norwich, UK). *E. coli* TunerDE3pLacI cells were transformed with the recombinant plasmids according to manufacturer's instructions. The expression was carried out in 1000 ml LB media growing cells at 37 °C until OD_600_ nm reached 0.4 to 0.6 and then induced at 16 °C for 48 h. The cells were harvested by centrifugation at 7000*g* for 10 min. The His-tagged proteins were purified by immobilized metal affinity chromatography (IMAC) and further purified by gel filtration (Superdex 75 and 200 columns) on an Akta system (GE Health Care Life Sciences, Little Chalfont, UK). Protein purification was assessed by standard SDS–polyacrylamide gel electrophoresis using the NuPAGE Novex 4–12% Bis–Tris (Life Technologies, Paisley, UK). Protein concentration was measured with a NanoDrop (Thermo Scientific, Wilmington, USA) and using the extinction coefficient calculated by ProtParam (ExPASy-Artimo, 2012) from the peptide sequence.

### Glycan microarrays

Three concentrations (5, 50 and 200 µg/ml) of recombinant His6-tagged E1_10125 D221A mutant were screened for binding to Core H glycan microarray glycans at the Consortium for Functional Glycomics (CFG).

### STD NMR experiments

An Amicon centrifuge filter unit with a 10 kDa MW cutoff was used to exchange the protein in 25 mM *d*_19_-2,2-bis(hydroxymethyl)-2,2′,2″-nitrilotriethanol pH* 7.4 (uncorrected for the deuterium isotope effect on the pH glass electrode) D_2_O buffer and 50 mM NaCl. All the ligands were dissolved in 25 mM *d*_19_-2,2-bis(hydroxymethyl)-2,2′,2″-nitrilotriethanol pH* 7.4, 50 mM NaCl. A concentration of 50 µM was used for the enzyme and 2 mM for the ligands. The STD NMR spectra were performed on a Bruker Avance 800.23 MHz at 278 K. The on- and off-resonance spectra were acquired using a train of 50 ms Gaussian selective saturation pulses using a variable saturation time from 0.5 to 5 s, for binding epitope mapping determination (STD build-up curves). Residual protein resonances were filtered out using a T_2_ filter of 40 ms. All the spectra were performed with a spectral width of 10 kHz and 32768 data points using 256 or 512 scans. Binding epitope mappings were obtained by determining the initial slopes (${\text{STD}}_{0}$) calculated by performing a least-squares fitting of the following mono-exponential curve:

$${\text{STD}}\left( {t_{{{\text{sat}}}} } \right) = {\text{STD}}_{{\max}} \left( {1 - \exp \left( { - k_{{{\text{sat}}}} t_{{{\text{sat}}}} } \right)} \right),$$

where ${\text{STD}}\left( {t_{{{\text{sat}}}} } \right)$ is the STD intensity for a saturation time, $t_{{{\text{sat}}}}$, ${\text{STD}}_{{\max}}$ is the maximum STD intensity and $k_{{{\text{sat}}}}$ is the rate constant for saturation transfer. In the limit, $t_{{{\text{sat}}}} \to 0$:

$${\text{STD}}_{0} = {\text{STD}}_{{\max}} k_{{{\text{sat}}}} .$$

Importantly, ${\text{STD}}_{0}$ gives a value that is independent of any relaxation or rebinding effects, allowing for a more accurate binding epitope. The value of ${\text{STD}}_{0}$ was then normalized against the proton with the largest intensity to give values in the range of 0–100%, which were then mapped onto the ligand structure to give the corresponding binding epitope mapping.

### X-ray crystallography

The E1_10125 fucosidase His tag was removed using 3C-protease overnight at 4 °C at a mass ratio of 20:1, the His tag and 3C-protease was then removed by passing the sample over a nickel sepharose column. The final crystallization condition was 0.2 M magnesium chloride, 25% PEG 3350, 0.1 M bis–tris pH 5.5, 10 mM 2-Fucosyllactose  (2′FL). Sitting drop vapour diffusion crystallization experiments of E1_10125 or E1_10125 D221A were set up at a concentration of 20 mg/ml and monitored using the VMXi beamline at Diamond Light Source [32]. Crystals were cryoprotected using the crystallization condition with the addition of 15% ethylene glycol. Wild-Type and D221A E1_10125 mutant diffraction experiments were performed at Diamond Light Source on beamlines I04 (wavelength 0.9795 Å) and I03 (wavelength 0.9763 Å), respectively. The data were processed with Xia2 making use of aimless, dials and pointless [33–36]. The data were phased using PHASER using pdb 4OUE as a molecular replacement model and refined using REFMAC [37] and Coot [38] within the CCP4 software environment [39]. The PDB REDO server [40] was used to optimize the refinement parameters and the model was validated using the Molprobity server [41].

### Molecular dynamics (MD) simulation and docking of sLeX into E1_10125 D221A

Docking calculations were run using the crystal structure of E1_10125 D221A mutant as it showed the highest resolution. First, a MD simulation was carried out to explore the flexibility of the side chains surrounding the fucose binding subsite and the whole binding site. The input coordinates for the simulation were produced by loading the X-ray coordinates into Schrödinger Maestro [42] and processed using the protein preparation wizard [43]. Protons were added to the structure and then all buffer ions, buffer and structurally non-essential water molecules were removed. PROPKA [44] was then used to predict the ionization state of polar residues at pH 7. The OPLS force field was then used to minimize the protein structure, converging heavy atoms to a threshold of 0.3 Å.

The system was then simulated using the Amber PMEMD software [45]. The system was solvated using TIP3P water, placed within a truncated octahedron buffered to 10 Å, to a net charge of zero using Na^+^ ions. The parameter set for the protein atoms and structural ions used was taken from the ff14SB and gaff force fields. The system was initially minimized with constraints of 20 kcal mol^−1^ Å^−2^ placed on solute atoms and then minimized a second time with no constraints placed on solute atoms. The system was then heated to a temperature of 300 K and raised to a pressure of 1 atm in two separate 500 ps steps under constrains of 20 kcal mol^−1^ Å^−2^ placed on solute atoms. Over the course of four 500 ps steps constrains were then released in 5 kcal mol^−1^ Å^−2^ increments. The system was then simulated over the course of 500 ns with a 2 fs time step, with frame sampling every 0.5 ns. The SHAKE algorithm [46] was used to constrain bonds involving hydrogen atoms. A Berendsen barostat and a Langevin thermostat with a 5 ps^−1^ collision frequency were used to maintain constant pressure and temperature, respectively. Non-bonding atom bond cutoff was set to 8 Å.

The trajectory file from the MD simulation was then clustered using *cpptraj* [47] to produce 20 average structures. The kmeans clustering algorithm within *cpptraj* was used with a random set of initial points. The clustering was calculated for every tenth frame and based on the distance between atoms measured using root-mean-square deviation of atomic positions (RMSD) without fitting structures to each other prior to calculating RMSD.

The 20 average structures were then imported into Schrödinger Maestro and processed using the protein preparation wizard (see above). Protons were replaced in the structure then all buffer ions and structurally non-essential water molecules introduced during MD simulation were removed. The OPLS force field was then used to minimize the protein structure, converging heavy atoms to a threshold of 0.3 Å. Protein structures showing a wide-open binding site were selected from the 20 average structures to be used for docking of the tetrasaccharide ligand. In order to perform the docking calculations, a cubic grid with a 10 Å × 10 Å × 10 Å inner box and a 30 Å × 30 Å × 30 Å outer box with the centroid placed at the middle of the binding site was generated. sLeX was built within Maestro and prepared using LigPrep [48] and low-energy conformers generated using MacroModel [49]. sLeX was then docked using Glide [50] with standard precision enhanced by 2 times without canonicalization and without sampling ring conformations.

### Isothermal titration calorimetry (ITC)

ITC experiments were performed using the PEAQ-ITC system (Malvern, Malvern, UK) with a cell volume of 200 µl. Prior to titration, protein samples (E1_10125D221A) were exhaustively dialyzed into 50 mM citrate buffer pH 6. The ligand was dissolved in the dialysis buffer. The cell protein concentration was 100 µM and the syringe ligand concentration was 2 mM for all ligands tested except 20 mM for Neu5Ac. Three controls with titrant (sugar) injected into the buffer, buffer injected to protein, buffer injected into buffer, were subtracted from the data. The analysis was performed using the Malvern software, using a single-binding site model. Experiments were carried out in triplicate.

### Activity assays and kinetics

The optimum pH of fucosidases was determined with 0.5 mM *p*NP-Fuc for all fucosidase tested apart for E1_10587 where 5 mM *p*NP-Fuc was used instead, in 50 mM citrate buffer (pH 3, 4, 5 and 6), 50 mM sodium phosphate buffer (pH 6, 7, 7.5 and 8) and 50 mM Tris buffer (pH 8.5 and 9.3). The final concentration of enzyme was 1 µM for ATCC_00842, 2 µM for E1_10180, 20 µM for E1_10125, 0.015 µM for ATCC_03833 and 10 µM for E1_10587. The reaction duration was optimized to measure the reaction rates under initial conditions. After incubation at 37 °C, the reaction was stopped by adding 50 µl of 1 M of sodium carbonate into 200 µl of reaction (200 µl of 1 M of sodium carbonate into 40 µl of reaction for E1_10587). The amount of fucose released was determined using a 96-well plate reader (BMG Labtech, Ortenberg, Germany) by measuring absorbance at 405 nm.

Kinetic studies against *p*NP-Fuc were performed in 50 mM citrate buffer at optimal pH (pH 6 for ATCC_00842, E1_10180, E1_10125, ATCC_03833; pH 5 for E1_10587) with increasing amounts of *p*NP-Fuc and a constant enzyme concentration at 37 °C. The series of *p*NP-Fuc concentrations were chosen to ensure that there were at least three points below and above the *K*_m_ value. The amount of enzyme was determined to fulfil free-ligand approximation, i.e. the enzyme concentration was linear with product formation. The reaction duration was optimized to measure the reaction rates under initial conditions. A standard curve was made with a range of *p*NP-Fuc from 0 to 140 µM and in the same experimental condition as the enzymatic reactions. Kinetic parameters were calculated based on the Michaelis–Menten equation using a non-linear regression analysis program (Prism 6, GraphPad, San Diego, USA). Kinetic parameters for E1_10587 were calculated based on the Michaelis–Menten equation:

$$\frac{1}{t}\ln \frac{{\left[ {S_{0} } \right]}}{{\left[ {S_{t} } \right]}} = - \frac{{\left[ {S_{0} - S_{t } } \right]}}{{K_{{\text{m}}} t}} + \frac{{V_{\max } }}{{K_{{\text{m}}} }}.$$

To determine the substrate specificity of the enzymes against fucosylated oligosaccharides (2′FL, 3FL, LeA and LeX), each enzyme was incubated with the substrate (0.1 mM) at 37 °C in 50 mM citrate buffer at optimal pH. For kinetic assays with E1_10125, 50 µM to 350 µM of LeX, 50 µM to 1400 µM of sLeX and 20 µM to 400 µM αGal-LeX was used. Higher concentrations of LeX or αGal-LeX were not used because they contained significant amount of l-fucose, which affected the accuracy of quantification. The enzyme in the reaction was 0.01 µM for LeX and sLeX, 1 nM for αGal-LeX. The time course of reaction used for LeX, sLeX and αGal-LeX was 9 min, 30 min and 20 min, respectively. The fucose released was quantified using the k-fucose kit (Megazyme, Wicklow, Ireland) combined with the diaphorase/resazurin assay [51]. Briefly, 40 µl of reaction mixed with 97 µl of mixed reagent (50 µl of dH_2_O, 20 µl of reaction buffer pH9.5, 5 µl of NADP + , 2 µl of l-fucose dehydrogenase suspension, 10 µl of 1 mM resazurin solution, 10 µl of 10U/ml solution of Diaphorase) and incubated at room temperature for 20 min before measuring the fluorescence of resorufin using a 96-wells plate reader (BMG Labtech, Ortenberg, Germany) with an excitation at 550 nm and an emission at 584 nm. One unit of activity was defined as the amount of enzyme needed to release 1 µmol of product per min under the conditions described above. The kinetic parameters of E1_10125 fucosidase against sLeX were calculated based on Michaelis–Menten equation using a non-linear regression analysis program (Prism 6, GraphPad, San Diego, USA). The curve of initial rate against substrate concentration for LeX and αGal-LeX was linear, which indicated that the substrate concentration was far below *K*_m_, therefore their *k*_cat_/*K*_m_ was estimated from the slope of the curve [52], *k* = (*k*_cat_/*K*_m_)[E]_0_ where *k* is the slope and [E]_0_ represents the enzyme concentration.

### LC–MS/MS analysis

10 µM of enzyme was incubated with a range of substrates including sLeA (5 µM), sLeX (5 µM), sialylated and de-sialylated human plasma *N*-glycans released from 0.5 µl human plasma, HRP (0.01 µg/µl), FA2G2 (5 ng/ µl), blood group A type II (5 µM) or blood group B type II (5 µM). Reactions (20 µl) were performed in 50 mM citrate buffer at pH 6 and 37 °C for 24 h. Reactions were then dried down using Savant SpeedVac centrifugal evaporator (Thermo Fisher, Wilmington, USA), labelled at the reducing end with procainamide using the glycan labelling kit with sodium cyanoborohydride reductant (Ludger, Oxford, UK) and purified using S-cartridges (Ludger, Oxford, UK) to remove the excess dye. The samples were dried by speed vacuum and resuspended in 50 µl of acetonitrile:water solvent. Then the reactions were injected onto a Waters ACQUITY UPLC Glycan BEH amide column (2.1 × 150 mm, 1.7 µm particle size, 130 Å pore size) at 40 °C on a Dionex Ultimate 3000 UHPLC instrument with a fluorescence detector (*λ*_ex_ = 310 nm, *λ*_em_ = 370 nm) coupled to a Bruker Amazon Speed ETD. A 50 mM ammonium formate solution pH 4.4 (Ludger, Oxford, UK) was used as mobile phase A and acetonitrile (Romil, UK) was used as mobile phase B. For the plasma samples, a 70 min gradient was used with mobile phase B from 76 to 51% from 0 to 53.5 min at a flow rate of 0.4 ml/min followed by mobile phase B from 51 to 0% from 53.5 min to 55.5 min at flow rate of 0. 2 ml/min, and 2 min stabilization, mobile phase B from 0 to 76% from 57.5 min to 59.5 min at a flow rate 0.2 ml/min, and then last for 6 min, from 65.5 min to 66.5 min, the flow rate was changed back to 0.4 ml/min and then equilibrated for 3.5 min. HRP samples used a 75 min gradient starting from 80 to 62% mobile B. A 70–62% gradient was used for FA2G2 glycan. For the shorter fucosyl-oligosaccharides, an 85 min gradient was used from 85 to 65% mobile B. The Amazon Speed was operated in the positive ion mode using the following settings: source temperature 180 °C; gas flow 4 L/min; capillary voltage, 4500 V; ICC target, 200,000; maximum accumulation time, 50 ms; rolling average, 2; number of precursor ions selected, 3; scan mode, enhanced resolution; mass range scanned, 400 to 1700. Singly charged ions were excluded for CID except for HRP and fucosyl-oligosaccharide samples.

### Bioinformatics analyses

For sequence similarity networks (SSN) analysis, the sequences encoding GH29 and GH95 fucosidases were extracted from the Interpro database 66.0 (https://www.ebi.ac.uk/interpro/) after removing redundant sequences by CD-HIT Suite [53] (https://weizhong-lab.ucsd.edu/cdhit_suite/cgi-bin/index.cgi?cmd=cd-hit). Additional sequences included those corresponding to functionally characterized GH29 and GH95 from the CAZy database (www.cazy.org) as well as the *R. gnavus* E1 and ATCC29149 fucosidases (6 GH29 and 7 GH95). The amino acid sequences were then used to create SSN using the Enzyme Function Initiative-Enzyme Similarity Tool (EFI-EST) [54]. The network setting for GH29 and GH95 was made to combine proteins in one node sharing over 70% and 45% identity, respectively and nodes were linked by edges when their sequences shared over 40% (the e-value threshold was 10^94^) and 35% identity (the e-value threshold was 10^130^) for GH29 and GH95, respectively. The SSN data were visualized using Cytoscape 3.6 [55].

ProtParam (ExPASy) [56] was used to determine the length, molecular weight and theoretical pI of the fucosidases under study. LALIGN was used to do pairwise sequence alignment and obtain sequence similarities [57]. SignalP 5.0 Server was used to predict the presence and nature of signal peptides as well as cleavage sites [58]. The TMHMM Server v. 2.0 was used to predict the presence of transmembrane helices [59]. CW-PRED was used for the detection of LPXTG and LPXTG-like motif and thus the prediction of cell-wall proteins in Gram-positive bacteria [60]. PSORTb v3.0.2 was used for bacterial protein subcellular localization prediction [61].

## Results

### Sequence similarity network identified strain-specific fucosidases in *R. gnavus* ATCC 29149 and E1

**Fig. 2:**
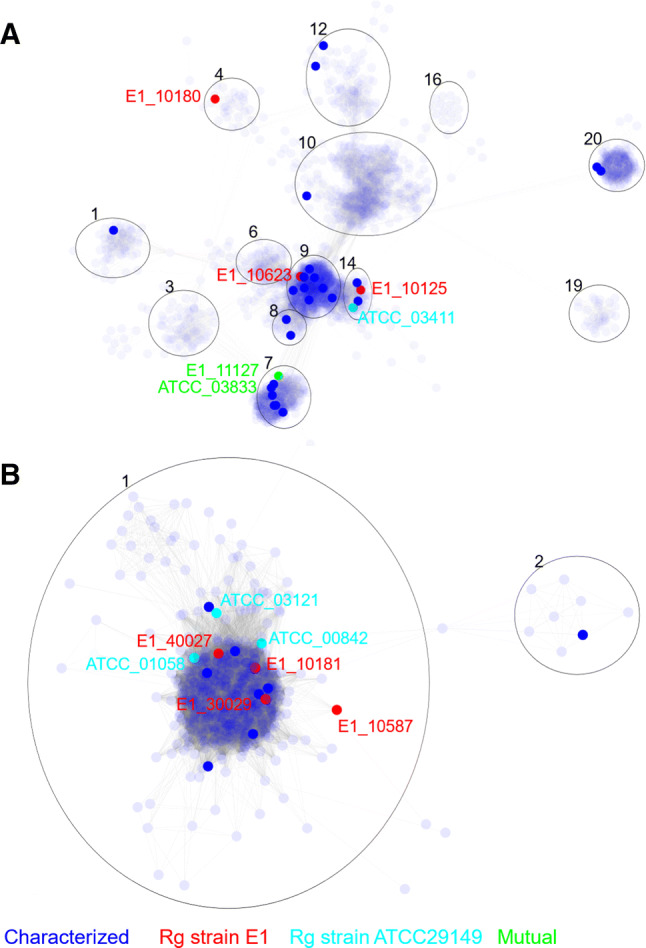
The distribution of *R. gnavus* GH29 and GH95 fucosidases based on SSN analysis. **a** Partial representation of SSN analysis of GH29 family containing fucosidases from *R. gnavus* E1 and ATCC29149 strains. **b** Representation of the SSN central cluster of GH95 family containing all GH95 from *R. gnavus* E1 and ATCC29149 strains. Blue node: sequences extracted from the CAZy database encoding functionally characterized enzymes. Red nodes sequences from *R. gnavus* E1 strain. Cyan nodes, sequences from *R. gnavus* ATCC29149 strain. Green nodes, sequences common to both *R. gnavus* E1 and ATCC29149 strains

The genome of *R. gnavus* E1 encodes 4 predicted GH29 fucosidases (named E1_10125, E1_10180, E1_10623 and E1_11127) and 4 GH95 fucosidases (named E1_10181, E1_10587, E1_30029, E1_40027), whereas *R. gnavus* ATCC 29149 encodes 2 GH29 (named ATCC_03411, ATCC_03833) and 3 GH95 fucosidases (named ATCC_00842, ATCC_01058, ATCC_03121) (for detailed information, see Table S2). A sequence similarity network (SSN) analysis was conducted to identify putative functional relationships between GH29 or GH95 fucosidases from *R. gnavus* strains and related protein sequences (Fig. 2). The SSN analysis covered 6736 amino acid sequences from the GH29 family from Interpro database 66.0 and CAZy (www.cazy.org/GH29_characterized.html) and 825 GH95 sequences from Interpro IPR027414 (including 8 sequences from functionally characterized GH95 www.cazy.org/GH95_characterized.html).

For GH29, 5318 representative nodes in the SSN analysis were separated into 44 clusters according to their sequence similarity which may indicate a similar biochemical function and ligand specificity (Fig. S1A). GH29 fucosidases from *R. gnavus* E1 and ATCC 29149 strains were found in cluster 4 (E1_10180), cluster 7 (ATCC_03833 and E1_11127), cluster 9 (E1_10623) and cluster 14 (E1_10125 and ATCC_03411) (Fig. 2a). E1_10125 shares 94.5% sequence similarity with ATCC_03411, whereas ATCC_03833 is 99.8% similar to E1_11127. The catalytic domain of E1_10623 was 62.9% similar to Blon_2336 from *Bifidobacterium longum* subsp. infantis ATCC 15697 [62]. Based on this analysis, E1_10125, E1_10180 and ATCC_03833 were chosen as representatives of *R. gnavus* GH29 fucosidases for functional characterization.

The GH95 family includes fewer sequences and functionally characterized proteins (www.cazy.org). The SSN analysis of GH95 fucosidases led to the identification of 825 nodes, 627 of which were found in the cluster 1 (Fig. 2b). All GH95 fucosidases from *R. gnavus* E1 and ATCC 29149 strains fall within the same cluster. Based on the pre-screening for expression (not shown), ATCC_00842 and E1_10587, sharing 60% sequence similarity, were selected as representative GH95 fucosidases of *R. gnavus* ATCC 29149 and E1, respectively for further characterization.

### *R. gnavus* fucosidases from GH29 and GH95 families display novel substrate specificities

The genes encoding the selected GH29 and GH95 fucosidases from *R. gnavus* ATCC 29149 and E1 strains were heterologously expressed in *E. coli* and the His6-tag recombinant proteins purified by immobilized metal ion affinity chromatography and gel filtration (see Materials and Methods for details). *E. coli* Tuner DE3 pLacI strain was chosen as heterologous host as it does not display any endogenous β-galactosidase activity (due to the deletion of the LacZ gene) that may interfere with the enzymatic characterization of the recombinant enzymes. The activity of the purified enzymes was first screened against the synthetic substrate *p*NP-Fuc. The optimum pH of all fucosidases tested, determined using *p*NP-Fuc, was found to be pH 6 apart for E1_10587, which was pH 5 (Fig. S2).

The kinetic parameters were determined at the optimum pH by calculating the initial rate of reaction with increasing *p*NP-Fuc concentrations. Fucosidase ATCC_03833 showed the highest catalytic efficiency with a *k*_cat_ of 83.6 s^−1^ and a *K*_m_ of 179.1 µM (Table 1). These values are consistent with fucosidases belonging to GH29 subfamily A [15, 17, 63, 64]. Fucosidases ATCC_00842 and E1_10180 also showed activity against *p*NP-Fuc, but their high *K*_m_ suggest that *p*NP-Fuc is not a good ligand for these enzymes. Fucosidase E1_10125 displays the lowest activity against *p*NP-Fuc of the characterized GH29 subfamily fucosidases as shown by its low *k*_cat_ of 1.8 10^–3^ s^−1^ [15, 20–22, 65, 66]. E1_10587 was merely active on *p*NP-Fuc as also reported for other GH95 fucosidases [21].

**Table 1:** Kinetic parameters of *R. gnavus* fucosidases towards *p*NP-Fuc

	E1_10125	E1_10180	ATCC_03833	ATCC_00842	E1_10587^a^
Catalytic efficiency (s^−1^ M^−1^)	7.61	16.91	4.67 ×10^5^	28.14	0.72
*K*_m_ (µM)	237.9 ± 39.69	ND	179.1 ± 28.77	2.96 × 10^3^ ± 3.63 × 10^2^	1.50 × 10^4^
*k*_cat_ (s^−1^)	1.8 10^–3^ ± 8.8 10^–5^	ND	83.6 ± 2.97	0.0832 ± 0.0032	0.0108

*ND* could not be determined (using concentrations up to 20 mM *p*NP-Fuc)

^a^Kinetic parameters were determined from the progress curve

Next, the substrate specificity of the recombinant fucosidases was tested on a range of fucosylated oligosaccharides. The specific activity was determined based on fucose release against 2′FL (Fucα1,2Galβ1,4Glc), 3FL (Galβ1-4[Fucα1-3]Glc), Lewis A (LeA, Galβl-3[Fucα1-4]GlcNAc) and Lewis X (LeX, Galβ1-4[Fucα1-3]GlcNAc) (Table 2). From this analysis, fucosidases E1_10125 and E1_10180 showed substrate specificity towards α1,3/4 fucosylated linkages while fucosidases ATCC_00842 and ATCC_03833 showed preference for α1,2 linkages (Table 2). E1_10587 showed lower activity against all tested substrates, therefore, it was not possible to assess its substrate specificity.

**Table 2:** Specific activity of *R. gnavus* fucosidases towards fucosylated oligosaccharides

Specific activity (U/µmol)				
	2′FL	3FL	LeA	LeX
E1_10125	NS	63.23 ± 2.03	83.88 ± 2.56	114.72 ± 1.76
E1_10180	NS	0.119 ± 0.001	0.45 ± 0.02	0.36 ± 0.01
ATCC_03833	5.47 ± 0.28	4.82 10^–2^ ± 7.09 10^–4^	0.069 ± 0.003	ND
ATCC_00842	1.13 10^4^ ± 3.06 10^2^	15.96 ± 0.67	NS	NS
*E1_10587*	NS	NS	NS	NS

*ND* not detected under experimental conditions, *NS* not significant, less than 0.01 U/µmol

**Fig. 3:**
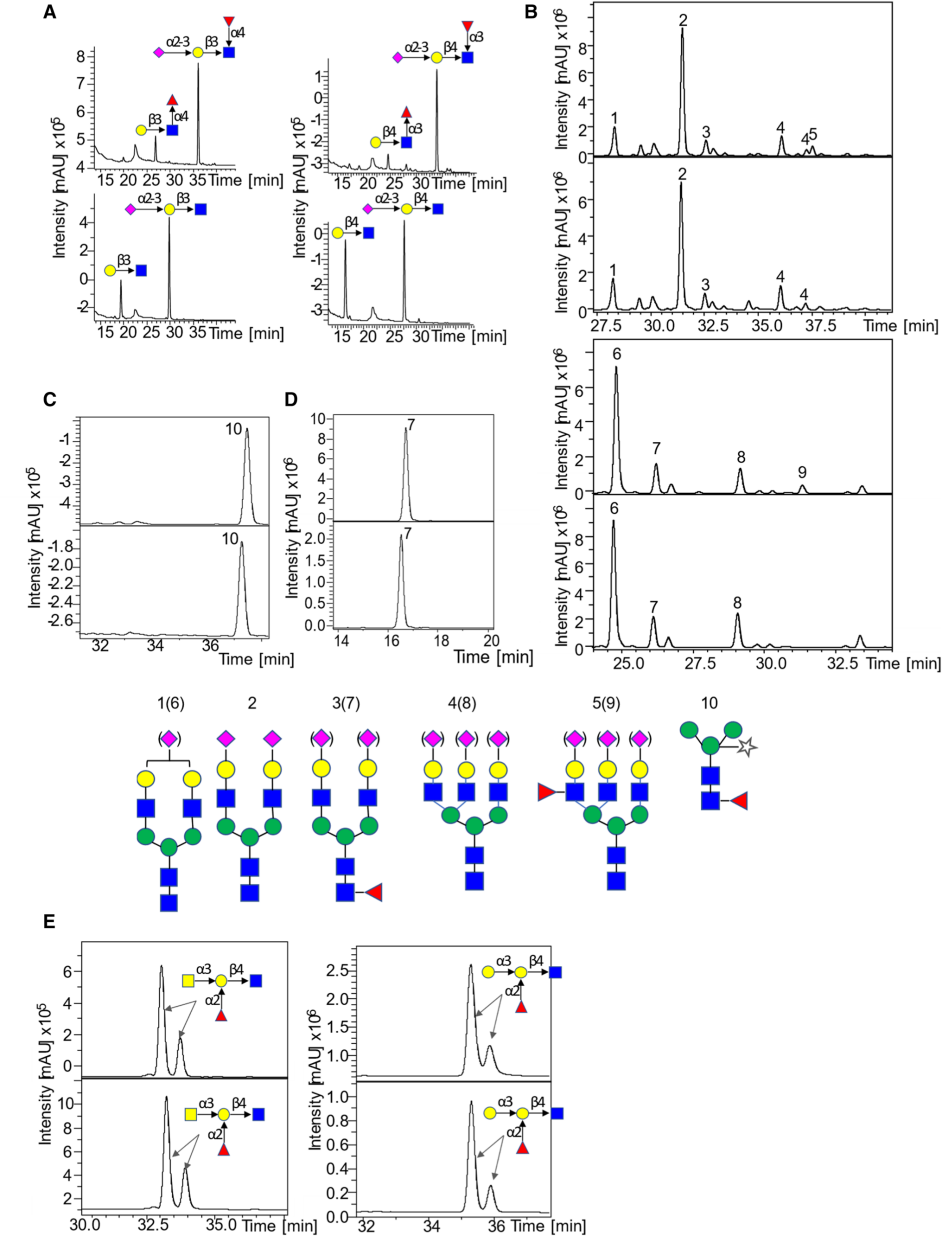
LC–MS/MS analysis of *R. gnavus* GH29 fucosidase E1-10125 towards various fucosylated substrates. **a** LC–MS/MS analysis of the products released from the enzymatic reaction of E1-10125 with LeA (left) and LeX (right), the upper graph is the negative control and the lower one corresponds to the enzymatic reaction. **B** LC–MS analysis of the products released from the enzymatic reaction of E1-10125 with sialylated (upper) and desialylated (lower) human plasma. The negative controls are showed on top of the enzymatic reactions. **c** LC–MS analysis of the products released from the enzymatic reaction of E1-10125 with HRP (core α1,3-fucose) (lower). The negative control is shown in the upper panel. D. LC–MS analysis of the products released from the enzymatic reaction of E1-10125 with FA2G2 (α1,6-fucose) (lower). The negative control is shown in the upper panel. **e** LC–MS analysis of the products released from the enzymatic reaction of E1-10125 with blood group A type II (left) and blood group B type II (right) (upper). The negative control is shown in the upper panel

The activity of the recombinant fucosidases was further tested on more complex oligosaccharides and glycoproteins including sialyl Lewis X (Neu5Acα2-3Galβ1-4[Fucα1-3]GlcNAc, sLeX), sialyl Lewis A (Neu5Acα2-3Galβl-3[Fucα1-4]GlcNAc, sLeA), sialylated or desialylated human plasma *N*-glycans, horseradish peroxidase *N*-glycans containing core α-1,3-fucose (HRP), blood group A type II, blood group B type II and α-1,6-fucosylated biantennary *N*-glycan (FA2G2) and the products of the reactions analyzed by LC–MS/MS (Table S3). Interestingly, this screening revealed that E1_10125 was active against α-1,3- and α-1,4-fucosylated substrates presenting a terminal sialic acid modification. The chromatograms clearly showed the appearance of peaks corresponding to Neu5Acα2-3Galβl-3GlcNAc and Neu5Acα2-3Galβ1-4GlcNAc and the disappearance of the peaks corresponding to sLeA and sLeX (Fig. 3a). The use of sialylated and de-sialylated *N*-glycans from human plasma confirmed the ability of E1_10125 to accommodate sialyl residues in terminal location of fucosylated *N*-glycans (Fig. 3b), as shown by the disappearance of the peak corresponding to fucosylated antennary *N*-glycan upon incubation with E1_10125. In contrast, no reaction product was detected when HRP glycans (core α1,3-fucose), FA2G2 (α1,6-fucose) or blood group antigens (α1,2-fucose) were used as substrates as shown by LC–MS traces (Fig. 3c–e).

### *R. gnavus* GH29 E1_10125 fucosidase can accommodate terminal sialic acid moieties in α1,3/4 antennary fucosylated substrates

**Fig. 4:**
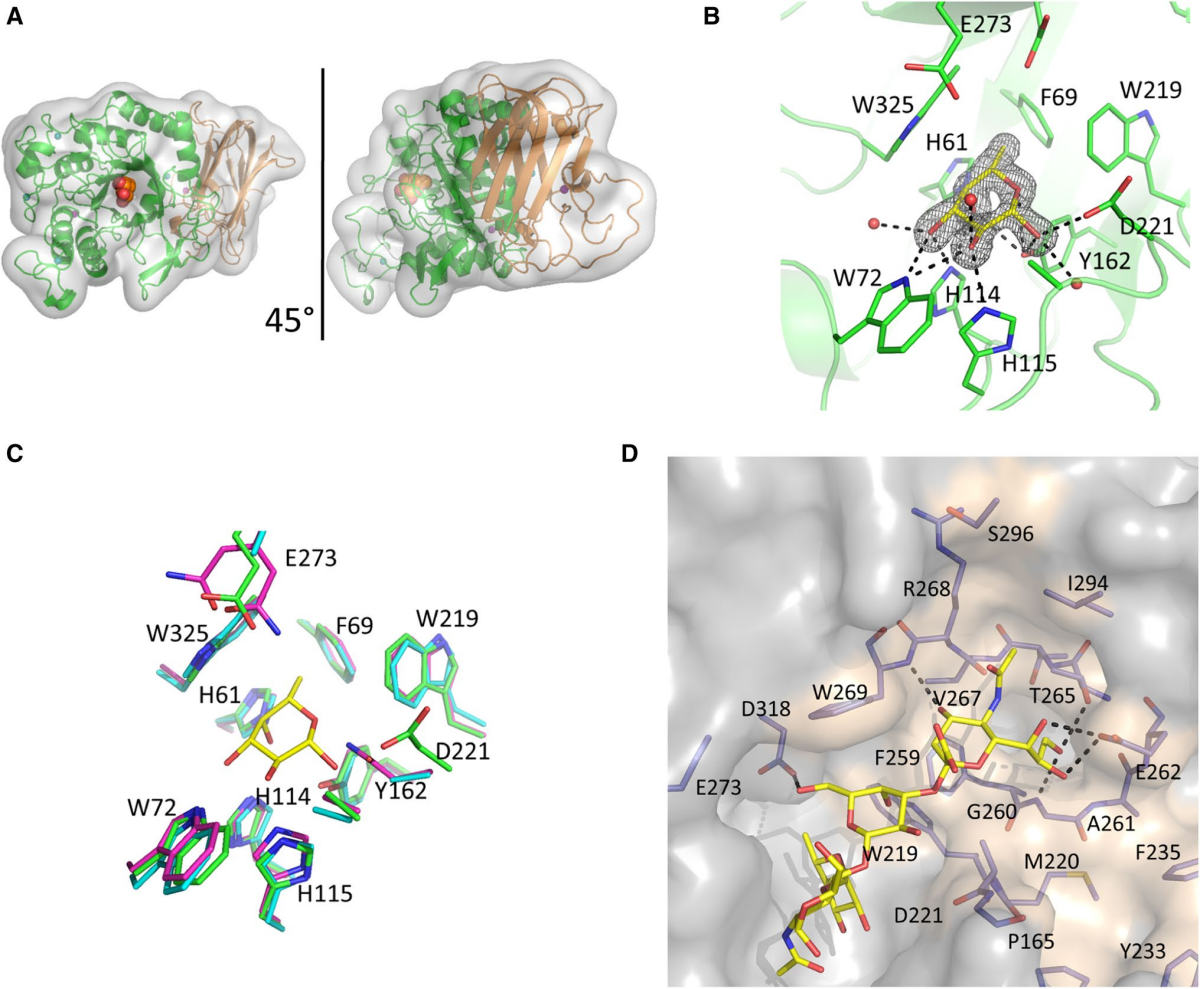
Crystal structure of *R. gnavus* GH29 fucosidase E1_10125. **a** Cartoon representation of E1_10125 fucosidase, the catalytic domain is coloured green and the proposed CBM is coloured orange. A fucose residue in a sphere representation indicates the location of the active site. The views are related by a 45° rotation around the *y* axis. **b** The E1_10125 fucose binding site. The β-anomer of fucose is shown in yellow with nearby active site residues shown in green. Black dashed lines indicate hydrogen-bonding interactions. Fo–Fc difference map density for the fucose residue is displayed as a black mesh, contoured at 2*σ*. **c** The fucose binding sites of E1_10125 (green), *S. pneumoniae* GH29 fucosidase (magenta), and *B. longum* subsp. infantis GH29 fucosidase (cyan) are aligned. Residue numbers refer to E1_10125. The binding site residues are conserved across the three structures and differences present at the D221 and E273 positions are catalytic mutants. Fucose bound in the E1_10125 is show in yellow for reference. **d** Model of the orientation and conformation of sLeX bound to *R. gnavus* E1_10125 proposed by MD simulations

To further investigate E1_10125 ligand specificity, the enzyme was crystallized in the presence of 2′FL, providing the crystal structure of the complex showing the β-fucose anomer bound in the active site (Fig. 4). Data collection and refinement statistics are detailed in Table 3. Electron density maps allowed modelling of residues 23–527. The enzyme consists of two distinct domains, a catalytic domain (PF001120) domain comprising residues 46–366 in N-terminal and a F5/8 Type C domain (PF00754) covering residues 385–526 in C-terminal (Fig. 4a). The catalytic domain displays a (α/β)_8_ which is typical of GH29 enzymes (www.cazy.org) whereas the type C-domains shows structural homology with carbohydrate binding module (CBM) belonging to CBM32 family (www.cazy.org). The macromolecular architecture is conserved with the recently solved GH29 fucosidase enzymes from *S. pneumoniae* (RMSD: 0.969  Å) [22] and *Bifidobacterium longum* subsp. infantis (RMSD: 1.128 Å) [67]. Residues 23–45 wrap around the C-terminal β-sandwich domain. The catalytic machinery sits in a cleft in the center of the N-terminal domain (Fig. 4b). By proximity to the bound fucose residue and homology to other fucosidases, Asp221 was identified as the catalytic nucleophile and Glu273 as the acid/base (amino acid numbering based on recombinant protein sequence) (Fig. 4b). Trp325 creates a CH–π interaction with the bound ligand and His61, Trp72, His114, His115 and Tyr162 provides additional hydrogen bonding interactions. Phe59 and Trp219 provide a hydrophobic pocket to accommodate the fucose C6 methyl group (Fig. 4b). The fucose binding site is conserved with the *S. pneumoniae* [22] and *B. longum* GH29 fucosidases [67] and is henceforth referred to as the − 1 subsite (Fig. 4c). In an attempt to obtain crystal structures in complex with the substrate, an active site mutant (E1_10125 D221A) was generated. However, following co-crystallization experiments with 2′FL, fucose was found bound in the active site, indicating residual fucosidase activity. Clear electron density was present for both α- and β-fucose anomers (Fig. S3A and S3B). It was not possible to obtain crystals of E1_10125 D221A in the absence of 2′FL. Attempts were made to displace the fucose molecule with other fucosylated ligands; however, these were unsuccessful, perhaps due to the substrate binding site being present at a tightly packed interface between two symmetry related protein molecules. No crystal structures could be obtained with other substrates tested including l-fucose, Neu5Ac, 3FL, LeA, LeX and sLeX.

**Table 3:** Data collection and refinement statistics

Data set	WT—Fucose	D221A—Fucose
PDB identifier	6TR3	6TR4
Data collection		
Space group	C2	P1
Cell dimensions		
a, b, c (Å)	164.2, 48.8, 132.1	49.8, 74.1, 76.5
α, β, γ (°)	90.0, 151.1, 90.0	82.5, 80.4, 70.4
Resolution (Å)	63.90–1.70 (1.73–1.70)	69.54–1.45 (1.47–1.45)
*R*_merge_	0.08 (0.59)	0.04 (0.11)
*R*_meas_	0.10 (0.72)	0.06 (0.16)
I/σI	6.9 (1.1)	12.7 (3.4)
CC half	0.99 (0.58)	0.99 (0.96)
Completeness	99.1 (97.5)	92.7 (56.9)
Redundancy	3.1 (2.9)	2.0 (1.8)
Refinement		
Resolution (Å)	63.86–1.70	69.54–1.45
No. of reflections	55,403	166,349
*R*_work_/*R*_free_	0.174/0.227	0.141/0.161
No. of atoms		
Protein	4022	8134
Ligand/ion	30	48
Water	282	1507
B-factors		
Protein	25.2	11.5
Ligand/ion	39.0	6.7
Water	26.9	23.6
r.m.s.d		
Bond lengths (Å)	0.03	0.01
Bond angle (°)	2.8	1.4
Ramachandran statistics (%)		
Favoured	96	97
Outliers	0	0

Numbers in parenthesis refer to the highest resolution shell

Superimposition of the E1_10125 crystal structure with that of α-1,3/4-fucosidase from *B. longum* subsp. infantis D172A/E217A mutant complexed with lacto-N-fucopentaose II (pdb 3UET) [67] (Fig. S4C) or with *S. pneumoniae Sp*GH29C^T^ D171N/E215Q in complex with LeA (pdb 6ORF) (Fig. S4D) or with LeX (pdb 6OR4) [62] (Fig. S3E) indicate that there are unlikely to be E1_10125 interactions that form a distinct + 1 site (GlcNAc in LeA and LeX trisaccharide antigens). E1_10125 Trp269 is conserved with *S. pneumoniae* and *B. longum* fucosidases, maintaining the + 2 site. More specifically, Trp269 is likely to form a CH/π stacking interaction with the galactose ring, and Asp318 to form a hydrogen bond with the galactose C6 hydroxyl. In the E1_10125 crystal structure, adjacent to the proposed + 2 site is an open platform comprises primarily neutral and hydrophobic residues, which would accommodate Neu5Ac (Fig. S3F). This is in marked contrast to the *S. pneumoniae* and *B. longum* homologue structures where this region is partially occluded by incoming loops. Molecular modelling calculations were carried out to further support this hypothesis and to provide a model for the orientation of the sialic acid ring of sLeX when bound to E1_10125 fucosidase (Fig. 4d). Following 500 ns MD simulations of the E1_10125 D221A mutant and docking of the sLeX ligand, the molecular model showed that sialic acid ring sits in the neighboring subsite. Polar contacts are established between nearby residues, Glu262 and Trp269; in particular, and hydroxyl groups present at sialic acid C4, C8 and C9 positions. This analysis confirmed that the E1_10125 fucosidase enzyme shows an open binding site able to accommodate the sLeX ligand.

In the absence of a complex structure of E1_10125 with a fucosylated oligosaccharide and in order to test the hypothesis that the cavity could accommodate the sialic acid moiety, E1_10125 R268W and E1_10125 G260M mutants were produced in which these introduced side-chains are expected to block access to the cavity. The E1_10125 R268W mutant showed a complete loss of activity towards all substrates tested including *p*NP-Fuc, 2′FL, 3FL, blood group A type II, blood group B type II, LeX and sLeX (data not shown) whereas the E1_10125 G260M mutant showed a significant decrease in activity towards sLeX down to 28% activity while 76% activity remained towards LeX (Table S4), suggesting that the cavity is important to accommodate terminal modifications of the fucosylated substrates.

Glycan arrays were then used to further define the ligand and linkage specificity of E1_10125 (Fig. S5). The purified recombinant His6-tagged E1_10125 D221A inactive mutant was screened at three protein concentrations against the Core H glycan microarray glycans at the Consortium for Functional Glycomics (CFG). Among the 585 glycans screened on the microarray, significant RFU values (> 300) were obtained for 5 fucosylated glycans using the highest protein concentration. Glycan ID 389 with α-Gal-LeA epitope displayed the highest RFU value (1072 ± 47) followed by two glycans, ID 249 and ID 526, containing sLeX epitopes. This recognition pattern therefore suggests that E1_10125 could recognize fucosylated substrates with diverse terminal modifications at the reducing end.

**Fig. 5:**
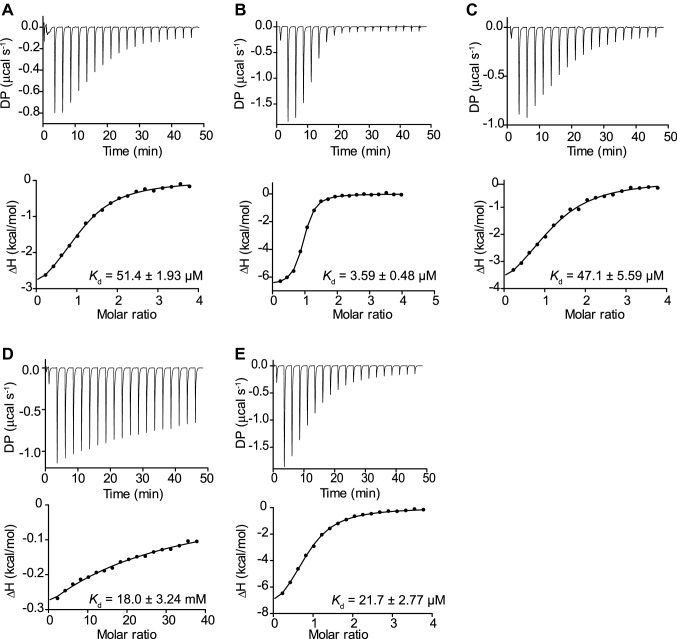
ITC isotherms of *R. gnavus* GH29 fucosidase E1_10125 binding to fucosylated ligands. **a** E1_10125 binding to LeX. **b** E1_10125 binding to sLeX. **c** E1_10125 binding to αGal-LeX. **d** E1_10125 binding to Neu5Ac. **e** E1_10125 binding to l-Fucose. *DP* differential power

In order to further test this hypothesis, ITC was used to determine the binding parameters of E1_10125 D221A mutant towards these ligands (Fig. 5 and Table S5). The enzyme bound to LeX with a *K*_d_ of 51.43 ± 1.93 μM (Fig. 5a) and to sLeX with a *K*_d_ of 3.59 ± 0.48 μM (Fig. 5b). Further, a *K*_d_ of 47.13 ± 5.60 μM was obtained when αGal-LeX (Fig. 5c) was used as a ligand whereas a *K*_d_ of 17.98 mM and 21.7 μM were obtained with the monosaccharides Neu5Ac or Fuc used as a control (Fig. 5d, e). To compare the substrate specificity among LeX, sLeX and αGal-LeX, the kinetic parameters were determined against these substrates (Table 4). E1_10125 showed strongest affinity to sLeX with a *K*_m_ of 163.1 µM and the presence of sialic acid or galactose on the non-reducing end of LeX significaly increased the catalytic efficienty up to 20-fold, consistent with the binding parameters (Table 4).

**Table 4:** Kinetic parameters of E1_10125 towards LeX, sLeX and aGal-LeX

	LeX	sLeX	αGal-LeX
Catalytic efficiency (s^−1^ M^−1^)	1416.67 ± 288.68	12,874.85 ± 1620.36	28,888.89 ± 2545.88
*K*_m_ (µM)	ND	163.1 ± 16.67	ND
*k*_cat_ (s^−1^)	ND	2.10 ± 0.07	ND

*ND* could not be determined under experimental conditions

**Fig. 6:**
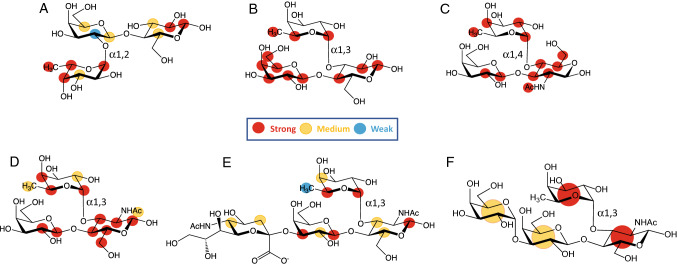
Binding epitope mapping from STD NMR spectroscopy depicting interactions of *R. gnavus* GH29 fucosidase E1-10125 with fucosylated oligosaccharides. Normalized saturation transfer intensities (0–100%) from STD NMR experiments mapped onto the chemical structures of **a** 2′FL, **b** 3FL, **c** LeA, **d** LeX, **e** sLeX and **f** αGal-LeX. Stronger normalized STD intensities correlate with closer ligand contacts with the surface of the protein in the bound state. Legend indicates normalized STD intensities: blue, 0–24%; yellow, 25–50%, red 51–100%. The enzyme intimately recognizes 3FL and LeA, whereas looser contacts are observed for 2′FL, LeX, sLeX and αGal-LeX. For the latter, a much higher degree of proton chemical shift overlapping implied lower binding epitope resolution and a normalized STD intensity value was assigned for each ring, as an average of the STD intensities of its isolated protons

To gain further structural insights into the unique ligand specificity of E1_10125, STD NMR studies [68] were conducted with E1_10125 D221A mutant in the presence of 2′FL, 3FL, LeA, LeX, sLeX and αGal-LeX (Fig. 6). Transfer of magnetization as saturation from the protein to the ligand was observed for all substrates tested, in agreement with the binding of E1_10125 to these substrates with medium-weak affinities (µM-mM). The enzyme intimately recognized the three sugar residues constituting 3FL and LeA (Fig. 6a, b) with no significant differences in their binding epitopes. 2′FL showed binding to E1_10125 D221A by STD NMR (Fig. 6c), but the main contacts were restricted to the fucose residue, whereas loose contacts were observed with the lactose disaccharidic moiety. The binding epitope of LeX also revealed a reduction in close contacts with the enzyme, particularly at the fucose residue. A comparison between 3FL (Fig. 6a) and LeX (Fig. 6d) supports an impact of the acetamido group (NHAc) at position 2 of GlcNAc on binding, which leads to changes in the contacts of the fucose ring with the protein in the bound state. This is highlighted by the reduction of the relative STD intensity of the methyl group at position 6 of this ring in the case of LeX. It is well established that the NHAc group in LeX limits the flexibility of the Fucα1–3GlcNAc linkage via steric hindrance with the adjacent fucose ring [69]. This leads to the observed changes in the contacts of the fucose ring in the bound state of LeX in comparison 3FL (Fig. 6d). These structural changes together with the advantageous reduction in entropy penalty upon binding expected for LeX due to the limited inter-glycosidic flexibility, are in good agreement with the observed differences in fucosidase activities (Table 2). The STD NMR results, in alignment with the activity assays and LC–MS/MS data, confirmed that the E1_10125 fucosidase shows a preference for α1-3/4 linkage (Fig. 3, Table 2), in which the fucose is linked at the reducing glucopyranose ring of the Gal β 1–3/4Glc(NAc) disaccharidic sequence. Interestingly, STD NMR revealed that the sialic acid moiety of sLeX makes contacts with the enzyme at C3 and C5 positions (Fig. 6e), suggesting that the sialic acid moiety is in part solvent exposed, and in part surrounded by residues at the protein surface, in agreement with the crystal structure showing a cavity that could accommodate such a sialic acid residue at the non-reducing end of the ligand. The molecular model (Fig. 4d) is also in excellent agreement with the experimental NMR data, as in this binding mode, protons at C3 and C5 are pointing towards the surface of the enzyme in the pocket. Likewise, in αGal-LeX, weak contacts were observed for the non-reducing αGal and βGal rings in the bound state, whereas fucose was the main ligand recognition moiety, followed by GlcNAc (Fig. 6f). Together, these data support the X-ray crystal structure that the binding pocket of E1_10125 could accomodate terminal residues although with a clear preference for sialic acid.

## Discussion

Fucose decorating glycan chains in HMOs or mucins contributes to shaping the composition of the gut microbiota in adults and infants. Previous studies in mice showed that the loss of the α-1,2-fucosyltransferase FUT2, and therefore fucosylated host glycans, leads to a decreased diversity and differences in intestinal microbial community [70–73], whereas an association between the composition of the intestinal microbiota and the ABO blood group or FUT2 secretor status was reported in humans [72, 74–77]. Human fetal mucins along the GI tract harbor a repertoire of *O*-glycans similar to HMOs [78, 79] which may also contribute to the differences in gut microbiota composition as compared to adults [80]. The ability to utilize fucosyllactose is a trait of early inhabitants of the human GI tract, such as *R. gnavus* [27] or various bifidobacteria species [81] as well as probiotic strains, such as *Lactobacillus casei* [82]. To access this nutrient source, gut bacteria have evolved to express a wide range of fucosidases with distinct ligand specificity, contributing to their fitness across nutritional niches [5, 6]. Furthermore, in its free form, fucose released by bacterial fucosidases may affect gut homeostasis. For example, *B. thetaiotaomicron* produces multiple fucosidases that cleave fucose from host glycans, resulting in high fucose availability in the gut lumen [83] which can then act as a signal to modulate the pathogenicity and metabolism of the pathogen enterohaemorrhagic *E. coli* (EHEC) [84].

Complexity in HMOs or mucins lies in the diversity of glycosidic bonds in these molecules, rendering a large number of potential combinations. Since fucosylation varies across and along the intestine and that fucosidase activity is dependent on the type of linkages present in the glycans or glycoconjugates, it is critical to understand the ligand specificity of the fucosidases encoded by major gut symbionts. Recently, the substrate specificities of two structure-solved GH29 fucosidases from *B. thetaiotaomicron* VPI-5482 were determined showing that the protein with locus tag BT 2970 belongs to GH29-A while BT 2192 belongs to GH29-B [15]. *R. gnavus* is a human gut symbiont of the infant and adult microbiota [23–25]. Here, we showed that *R. gnavus* strains encodes a range of fucosidases belonging to GH95 and GH29-A and GH29-B families with varied specificities, highlighting the versatility of fucosylated substrates they can access, such as those present in HMOs or intestinal mucins. The enzymatic characterization of *R. gnavus* fucosidases focused on previously uncharacterized bacterial fucosidases revealed that fucosidase ATCC_03833 belongs to GH29-A while E1_10125 belongs to GH29-B subfamily. GH29-B E1_10125 and E1_10180 showed strict substrate specificity towards α1,3/4 fucosylated linkages while fucosidases GH95 ATCC_00842 and GH29-A ATCC_03833 showed preference for α1,2 linkages, as reported for *B. bifidum* AfcA fucosidase [85]. *O*-glycan analyses of human fetal mucins showed that fucose is present in a large variety of terminal linkages, including blood group H as well as LeA (Galβ(1–3[Fucα1-4]GlcNAc), LeB (Fucα1-2Galβ1-3[Fucα1-4]GlcNAc), LeX (Galβ(1–4 [Fucα1-3]GlcNAc) and LeY (Fucα1-2Galβ1-4[Fucα1-3] GlcNAc) determinants [79]. The diversity of fucosidases may confer *R. gnavus* strains with an advantage in colonizing the infant gut [25].

In addition, we showed that E1_10125 could act on LeA and LeX even when the galactose moiety was linked to a sialic acid residue or other decorations. In particular, E1_10125 showed highest affinity towards sLeX. The ability to accommodate the sialic acid moiety, as also confirmed by STD NMR, appears to be enabled by an open region adjacent to the + 2 site (Gal residue in Le antigens), which  comprises residues Met220, Phe259, Gly260, Ala261 and Thr265 with additional stabilizing interactions likely to be provided by Trp269 and Glu262. This structural arrangement lacks the incoming loops present in the *S. pneumoniae* and *B. longum* GH29 enzymes [22, 67], supporting the unique specificity of *R. gnavus* E1_10125 fucosidase. Glycan array analyses suggested that E1_10125 could recognize fucosylated glycans with diverse terminal modifications as also supported by ITC showing binding of E1_10125 to both sialic acid and αGal linked to Gal of LeX. Extensive differences in the glycosylation profile of mucins occur along the GI tract, characterized by the presence of decreasing gradients of fucose and ABH blood group and increasing gradients of sialic acid from ileum to rectum [7, 86]. In human colonic mucin, more than 100 complex O-linked oligosaccharides were identified, mostly based on the core 3 structure with sialic acid at the 6-position of the GalNAc [9]. The most abundant components were -Gal-(Fuc)GlcNAc-3(NeuAc-6)GalNAcol, GalNAc-(NeuAc-)Gal-4/3GlcNAc-3(NeuAc-6)GalNAcol, GalNAc-3(NeuAc-6) GalNAcol and GlcNAc-3(NeuAc-6)GalNAcol [9]. The unusual specificity of E1_10125 may, therefore, contribute to the fitness and spatial adaptation of *R. gnavus* strains into the adult human GI tract [23, 24].

The specificities of *R. gnavus* fucosidases could be exploited for diagnostic assays. For example, changes in the abundance of antennary fucosylation in plasma *N*-glycans have been associated with diabetes [87, 88] and with colorectal cancer [89–91]. The quantitation of these low abundant antennary fucosylated glycans in the plasma *N*-glycome is complex because the structural diversity of its component glycans [92, 93] that rely on chromatographic platforms requiring extensive measurement time [91–95]. However, the recent technological advances integrating the use of fucosidases or other glycosidases and analysis on a MALDI-MS platform enabled identification and quantification of glycans of specific fucose isomers [96, 97]. The antennary fucosidase specificity reported in this work could therefore be used as a discriminatory tool to identify *N*-glycan biomarkers of diseases and as a valuable tool for the purpose of glycoprofiling biopharmaceutical glycoproteins.

## Electronic supplementary material

Below is the link to the electronic supplementary material.

Supplementary file1 (PPTX 9567 kb)

Supplementary file2 (DOCX 30 kb)
