# Supplementary material for: Fucosidases from the human gut symbiont Ruminococcus gnavus
Source: Cell Mol Life Sci. 2020 Apr 24;78(2):675–93. doi: 10.1007/s00018-020-03514-x (PMC7872956; doi:10.1007/s00018-020-03514-x)
Supplement: Supplementary file 1 — Supplementary file1 (PPTX 9567 kb) [file 18_2020_3514_MOESM1_ESM.pptx]

## Slide 1
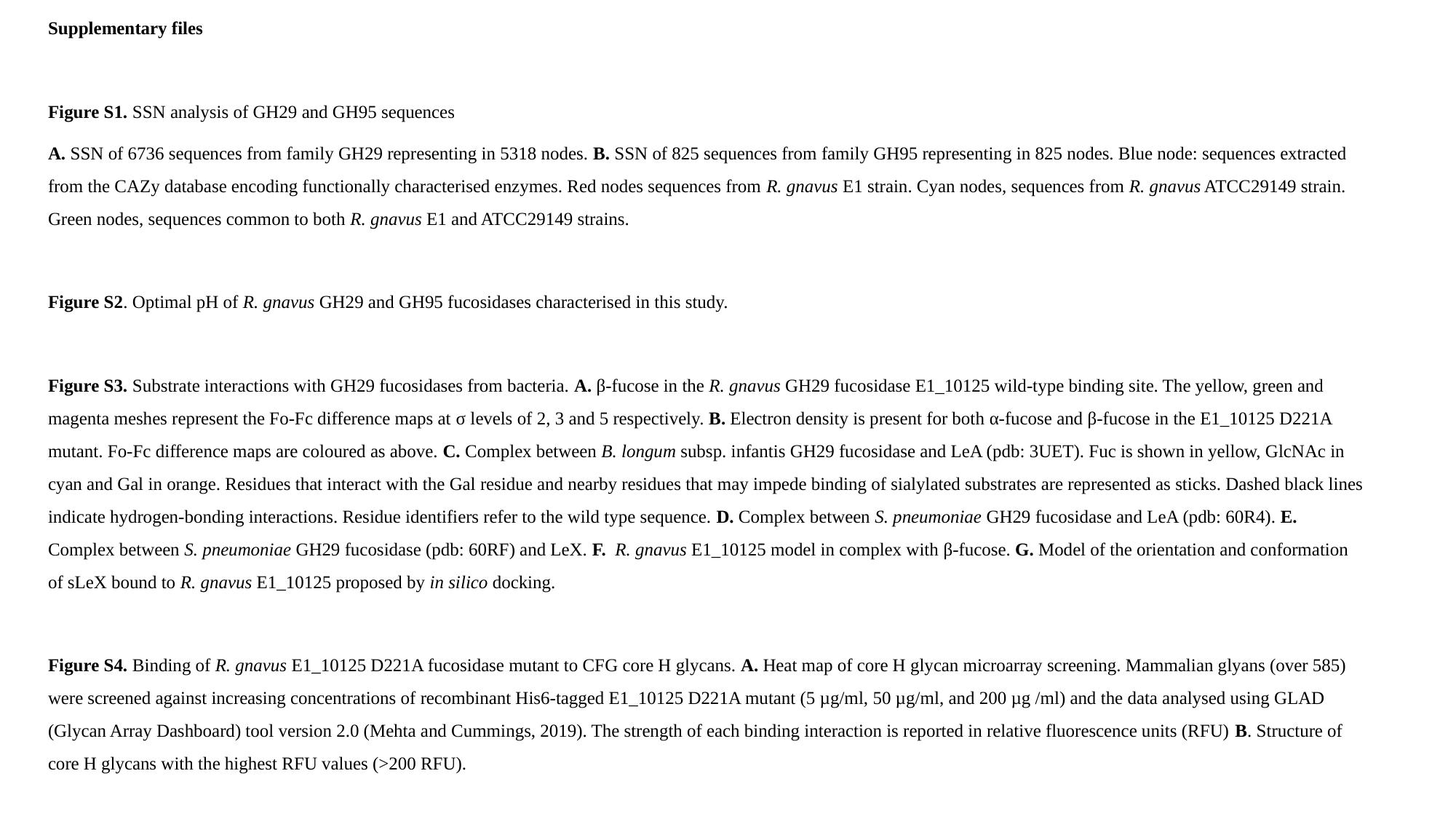

Supplementary files
Figure S1. SSN analysis of GH29 and GH95 sequences
A. SSN of 6736 sequences from family GH29 representing in 5318 nodes. B. SSN of 825 sequences from family GH95 representing in 825 nodes. Blue node: sequences extracted from the CAZy database encoding functionally characterised enzymes. Red nodes sequences from R. gnavus E1 strain. Cyan nodes, sequences from R. gnavus ATCC29149 strain. Green nodes, sequences common to both R. gnavus E1 and ATCC29149 strains.
Figure S2. Optimal pH of R. gnavus GH29 and GH95 fucosidases characterised in this study.
Figure S3. Substrate interactions with GH29 fucosidases from bacteria. A. β-fucose in the R. gnavus GH29 fucosidase E1_10125 wild-type binding site. The yellow, green and magenta meshes represent the Fo-Fc difference maps at σ levels of 2, 3 and 5 respectively. B. Electron density is present for both α-fucose and β-fucose in the E1_10125 D221A mutant. Fo-Fc difference maps are coloured as above. C. Complex between B. longum subsp. infantis GH29 fucosidase and LeA (pdb: 3UET). Fuc is shown in yellow, GlcNAc in cyan and Gal in orange. Residues that interact with the Gal residue and nearby residues that may impede binding of sialylated substrates are represented as sticks. Dashed black lines indicate hydrogen-bonding interactions. Residue identifiers refer to the wild type sequence. D. Complex between S. pneumoniae GH29 fucosidase and LeA (pdb: 60R4). E. Complex between S. pneumoniae GH29 fucosidase (pdb: 60RF) and LeX. F. R. gnavus E1_10125 model in complex with β-fucose. G. Model of the orientation and conformation of sLeX bound to R. gnavus E1_10125 proposed by in silico docking.
Figure S4. Binding of R. gnavus E1_10125 D221A fucosidase mutant to CFG core H glycans. A. Heat map of core H glycan microarray screening. Mammalian glyans (over 585) were screened against increasing concentrations of recombinant His6-tagged E1_10125 D221A mutant (5 µg/ml, 50 µg/ml, and 200 µg /ml) and the data analysed using GLAD (Glycan Array Dashboard) tool version 2.0 (Mehta and Cummings, 2019). The strength of each binding interaction is reported in relative fluorescence units (RFU) B. Structure of core H glycans with the highest RFU values (>200 RFU).

## Slide 2
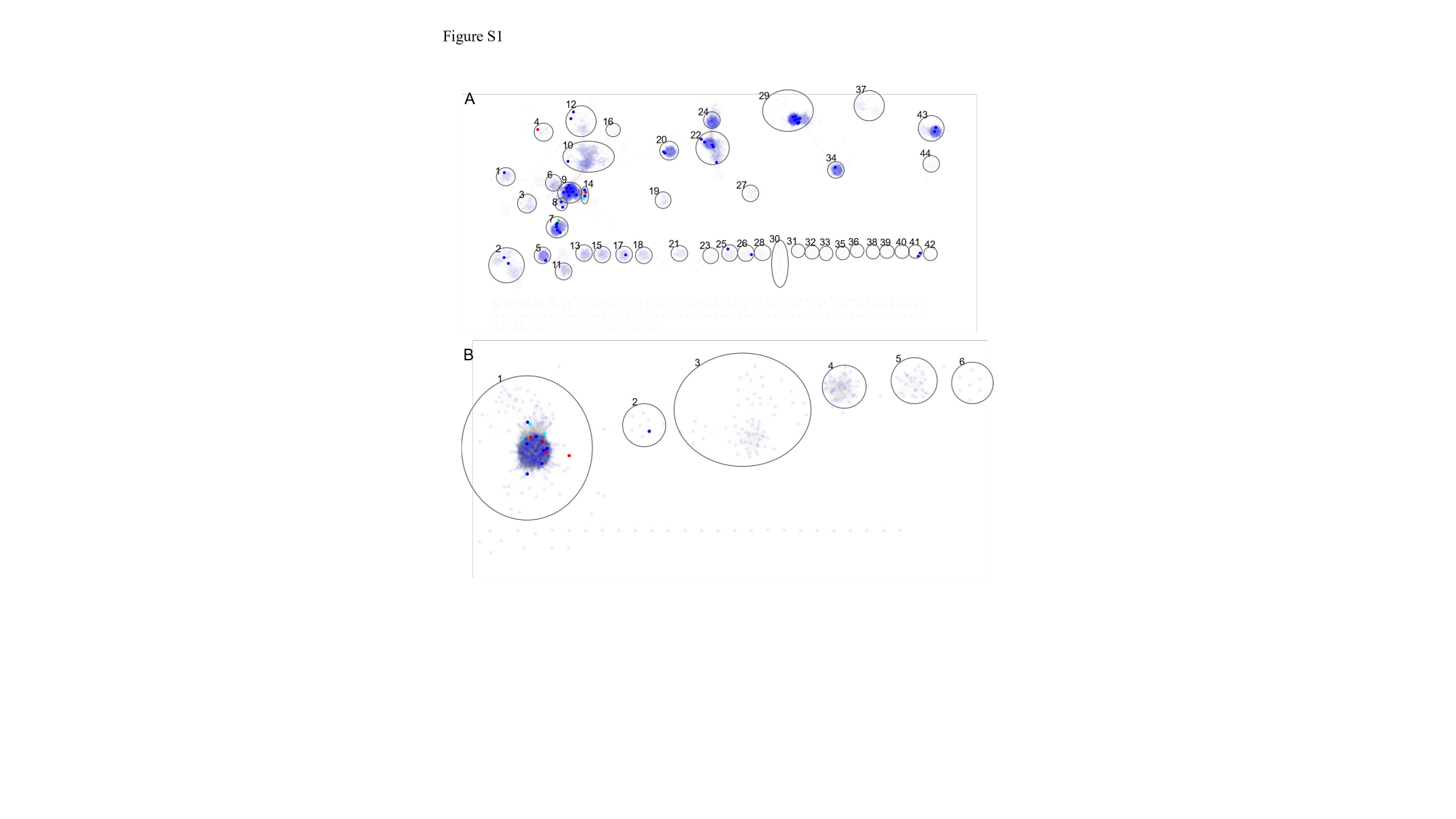

## Slide 3
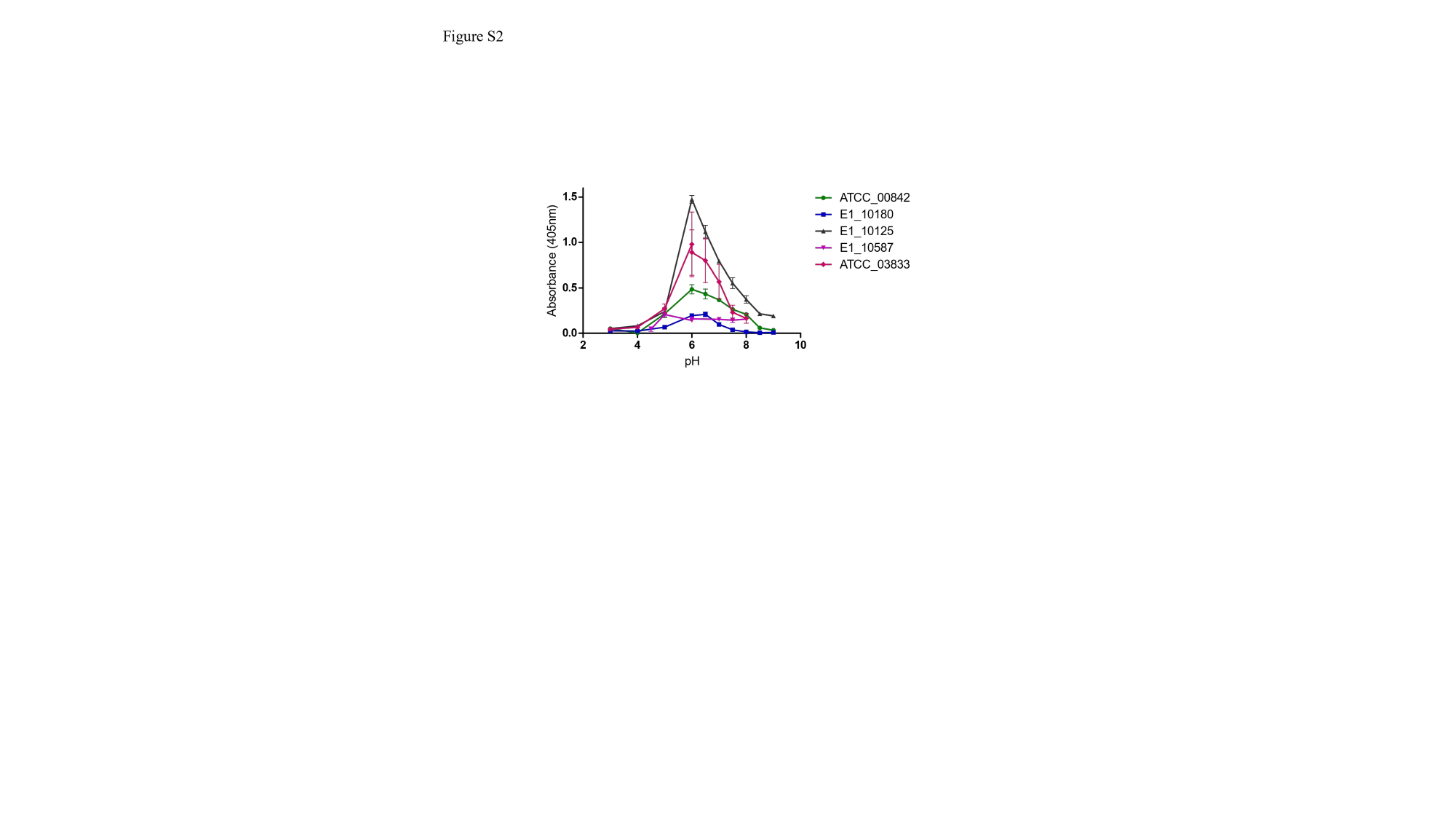

## Slide 4
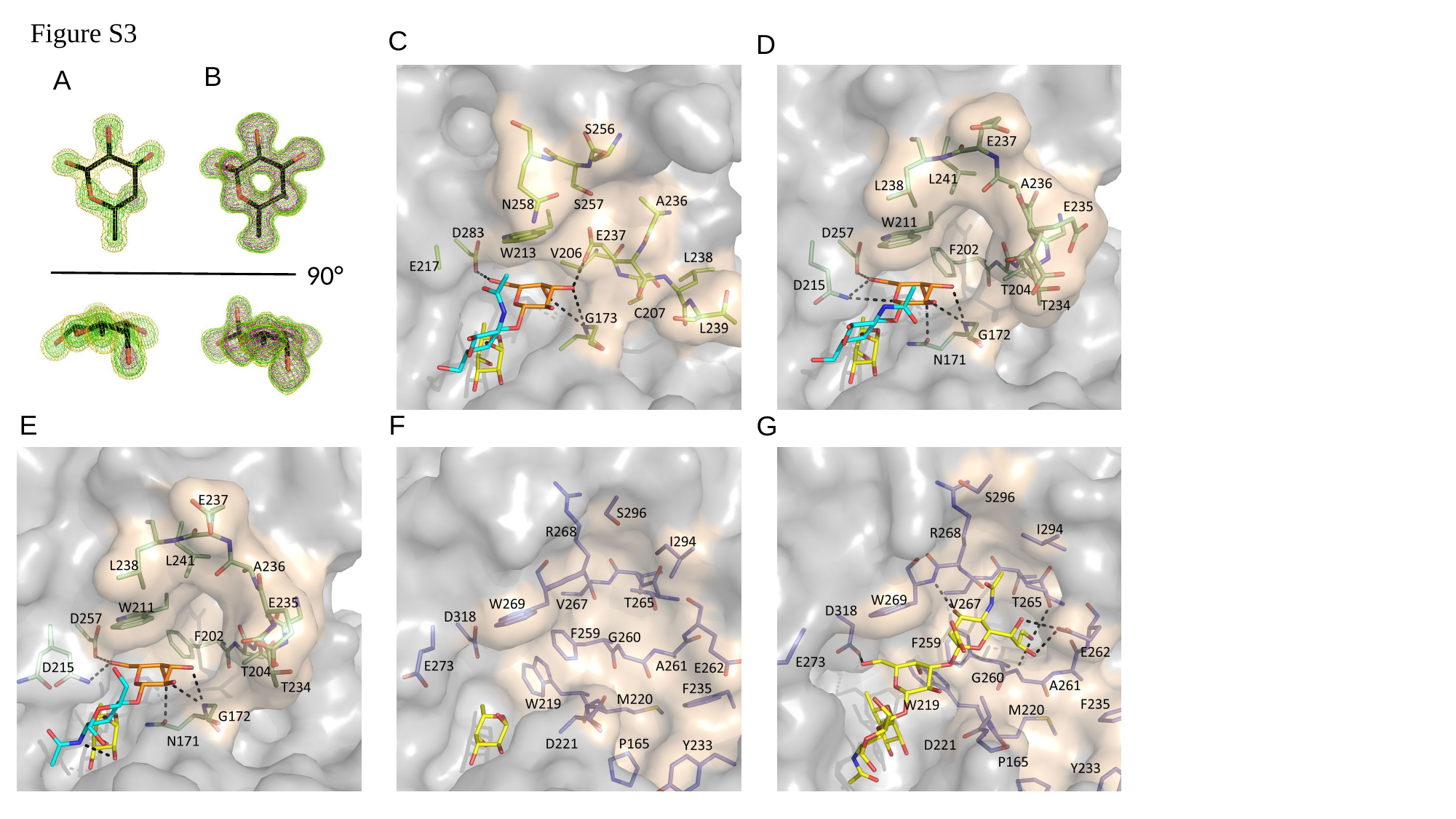

Figure S3
C
D
B
A
90°
E
F
G

## Slide 5
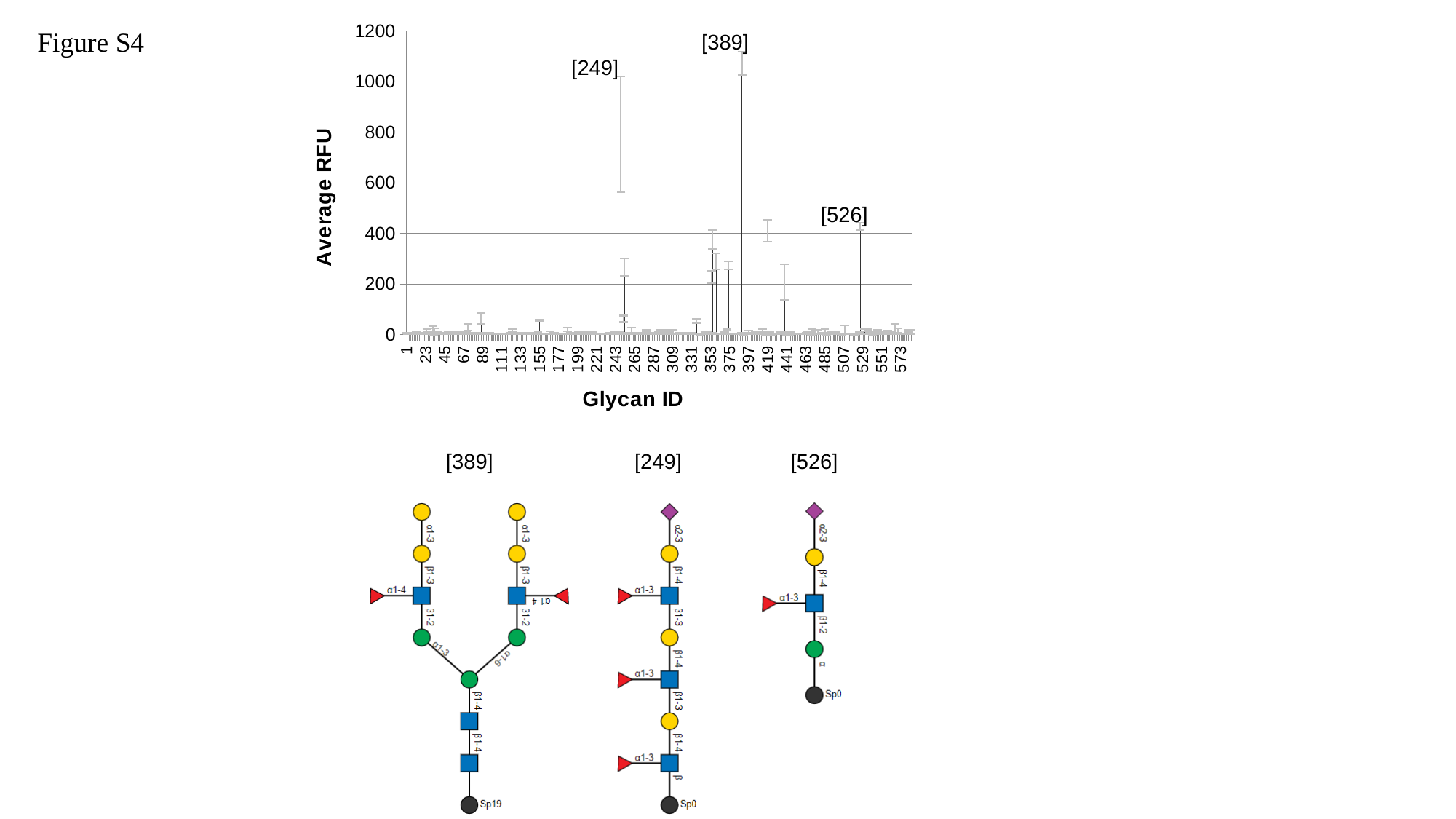

### Chart
| Category | Average RFU |
|---|---|
| 1 | 5.75 |
| 2 | 4.5 |
| 3 | 5.25 |
| 4 | 3.25 |
| 5 | 3.25 |
| 6 | 6.25 |
| 7 | 3.25 |
| 8 | 5.75 |
| 9 | 4.75 |
| 10 | 2.5 |
| 11 | 5.5 |
| 12 | 7.0 |
| 13 | 4.0 |
| 14 | 6.25 |
| 15 | 6.75 |
| 16 | 4.75 |
| 17 | 3.25 |
| 18 | 4.5 |
| 19 | 2.75 |
| 20 | 4.75 |
| 21 | 5.75 |
| 22 | 5.25 |
| 23 | 5.0 |
| 24 | 16.0 |
| 25 | 1.75 |
| 26 | 3.75 |
| 27 | 6.75 |
| 28 | 2.5 |
| 29 | 4.25 |
| 30 | 3.0 |
| 31 | 21.25 |
| 32 | 1.75 |
| 33 | 15.75 |
| 34 | 6.5 |
| 35 | 6.75 |
| 36 | 2.75 |
| 37 | 9.0 |
| 38 | 6.75 |
| 39 | 3.75 |
| 40 | 5.25 |
| 41 | 3.75 |
| 42 | 4.25 |
| 43 | 4.75 |
| 44 | 6.0 |
| 45 | 4.5 |
| 46 | 3.75 |
| 47 | 2.5 |
| 48 | 3.5 |
| 49 | 8.0 |
| 50 | 2.25 |
| 51 | 3.0 |
| 52 | 1.75 |
| 53 | 2.5 |
| 54 | 2.5 |
| 55 | 2.5 |
| 56 | 5.75 |
| 57 | 6.25 |
| 58 | 8.25 |
| 59 | 2.5 |
| 60 | 5.75 |
| 61 | 5.5 |
| 62 | 2.5 |
| 63 | 5.0 |
| 64 | 3.25 |
| 65 | 5.25 |
| 66 | 2.75 |
| 67 | 5.0 |
| 68 | 3.75 |
| 69 | 7.75 |
| 70 | 10.0 |
| 71 | 3.25 |
| 72 | 29.25 |
| 73 | 2.5 |
| 74 | 3.25 |
| 75 | 3.25 |
| 76 | 3.5 |
| 77 | 3.25 |
| 78 | 5.0 |
| 79 | 3.0 |
| 80 | 3.5 |
| 81 | 6.0 |
| 82 | 3.5 |
| 83 | 5.25 |
| 84 | 6.0 |
| 85 | 4.25 |
| 86 | 1.75 |
| 87 | 63.75 |
| 88 | 4.0 |
| 89 | 5.75 |
| 90 | 3.0 |
| 91 | 6.0 |
| 92 | 5.0 |
| 93 | 2.75 |
| 94 | 3.5 |
| 95 | 3.5 |
| 96 | 3.75 |
| 97 | 6.0 |
| 98 | 4.5 |
| 99 | 3.75 |
| 100 | 3.75 |
| 101 | 4.0 |
| 102 | 3.75 |
| 103 | 2.5 |
| 104 | 1.5 |
| 105 | 3.25 |
| 106 | 3.5 |
| 107 | 4.25 |
| 108 | 3.75 |
| 109 | 4.75 |
| 110 | 3.5 |
| 111 | 4.25 |
| 112 | 3.75 |
| 113 | 2.75 |
| 114 | 4.0 |
| 115 | 3.75 |
| 116 | 5.0 |
| 117 | 4.25 |
| 118 | 3.25 |
| 119 | 3.75 |
| 120 | 2.75 |
| 121 | 5.25 |
| 122 | 3.75 |
| 123 | 18.75 |
| 124 | 4.25 |
| 125 | 4.25 |
| 126 | 3.0 |
| 127 | 3.75 |
| 128 | 3.5 |
| 129 | 4.75 |
| 130 | 4.0 |
| 131 | 3.75 |
| 132 | 3.5 |
| 133 | 4.5 |
| 134 | 2.5 |
| 135 | 5.25 |
| 136 | 1.5 |
| 137 | 3.25 |
| 138 | 3.5 |
| 139 | 4.25 |
| 140 | 3.25 |
| 141 | 5.25 |
| 142 | 3.25 |
| 143 | 3.75 |
| 144 | 3.0 |
| 145 | 3.75 |
| 146 | 5.25 |
| 147 | 1.5 |
| 148 | 2.0 |
| 149 | 3.0 |
| 150 | 4.5 |
| 151 | 4.5 |
| 152 | 4.5 |
| 153 | 11.25 |
| 154 | 55.75 |
| 155 | 2.75 |
| 156 | 4.5 |
| 157 | 2.25 |
| 158 | 3.75 |
| 159 | 3.5 |
| 160 | 3.0 |
| 161 | 4.25 |
| 162 | 3.0 |
| 163 | 3.0 |
| 164 | 3.0 |
| 165 | 2.75 |
| 166 | 3.0 |
| 167 | 10.0 |
| 168 | 3.75 |
| 169 | 4.5 |
| 170 | 2.0 |
| 171 | 4.5 |
| 172 | 4.5 |
| 173 | 2.75 |
| 174 | 4.5 |
| 175 | 2.0 |
| 176 | 3.25 |
| 177 | 2.5 |
| 178 | 3.75 |
| 179 | 4.25 |
| 180 | 2.75 |
| 181 | 3.0 |
| 182 | 2.5 |
| 183 | 2.25 |
| 184 | 2.0 |
| 185 | 4.25 |
| 186 | 3.25 |
| 187 | 21.5 |
| 188 | 3.0 |
| 189 | 2.25 |
| 190 | 4.25 |
| 191 | 2.5 |
| 192 | 3.75 |
| 193 | 2.75 |
| 194 | 5.75 |
| 195 | 3.75 |
| 196 | 5.25 |
| 197 | 3.0 |
| 198 | 3.5 |
| 199 | 4.5 |
| 200 | 7.5 |
| 201 | 8.75 |
| 202 | 5.75 |
| 203 | 4.5 |
| 204 | 4.25 |
| 205 | 3.0 |
| 206 | 4.0 |
| 207 | 5.0 |
| 208 | 4.5 |
| 209 | 4.0 |
| 210 | 9.0 |
| 211 | 2.0 |
| 212 | 2.75 |
| 213 | 3.75 |
| 214 | 3.75 |
| 215 | 4.25 |
| 216 | 3.25 |
| 217 | 11.75 |
| 218 | 4.25 |
| 219 | 4.5 |
| 220 | 4.25 |
| 221 | 4.0 |
| 222 | 3.5 |
| 223 | 2.25 |
| 224 | 4.0 |
| 225 | 2.5 |
| 226 | 3.25 |
| 227 | 3.75 |
| 228 | 4.25 |
| 229 | 4.25 |
| 230 | 3.75 |
| 231 | 1.75 |
| 232 | 3.0 |
| 233 | 2.0 |
| 234 | 2.0 |
| 235 | 5.0 |
| 236 | 3.75 |
| 237 | 5.75 |
| 238 | 4.0 |
| 239 | 6.75 |
| 240 | 2.5 |
| 241 | 11.5 |
| 242 | 3.75 |
| 243 | 4.75 |
| 244 | 2.0 |
| 245 | 4.25 |
| 246 | 4.0 |
| 247 | 3.75 |
| 248 | 9.75 |
| 249 | 792.0 |
| 250 | 6.5 |
| 251 | 6.75 |
| 252 | 63.25 |
| 253 | 267.5 |
| 254 | 3.75 |
| 255 | 3.5 |
| 256 | 2.25 |
| 257 | 3.25 |
| 258 | 4.5 |
| 259 | 4.75 |
| 260 | 2.5 |
| 261 | 17.0 |
| 262 | 1.75 |
| 263 | 5.0 |
| 264 | 2.75 |
| 265 | 4.0 |
| 266 | 5.5 |
| 267 | 4.25 |
| 268 | 4.0 |
| 269 | 4.5 |
| 270 | 5.25 |
| 271 | 3.75 |
| 272 | 3.25 |
| 273 | 5.25 |
| 274 | 2.25 |
| 275 | 3.5 |
| 276 | 3.25 |
| 277 | 3.5 |
| 278 | 15.75 |
| 279 | 3.75 |
| 280 | 3.25 |
| 281 | 4.75 |
| 282 | 3.5 |
| 283 | 5.5 |
| 284 | 4.0 |
| 285 | 2.75 |
| 286 | 8.25 |
| 287 | 5.5 |
| 288 | 5.5 |
| 289 | 5.75 |
| 290 | 4.75 |
| 291 | 3.0 |
| 292 | 8.75 |
| 293 | 7.25 |
| 294 | 12.75 |
| 295 | 16.5 |
| 296 | 2.25 |
| 297 | 3.0 |
| 298 | 2.5 |
| 299 | 2.75 |
| 300 | 3.0 |
| 301 | 3.75 |
| 302 | 3.0 |
| 303 | 4.75 |
| 304 | 15.25 |
| 305 | 5.25 |
| 306 | 2.25 |
| 307 | 4.0 |
| 308 | 3.5 |
| 309 | 11.75 |
| 310 | 3.75 |
| 311 | 3.0 |
| 312 | 3.75 |
| 313 | 2.5 |
| 314 | 3.75 |
| 315 | 2.5 |
| 316 | 5.5 |
| 317 | 2.75 |
| 318 | 4.5 |
| 319 | 6.75 |
| 320 | 2.25 |
| 321 | 2.5 |
| 322 | 3.5 |
| 323 | 3.25 |
| 324 | 1.5 |
| 325 | 7.75 |
| 326 | 2.25 |
| 327 | 3.25 |
| 328 | 5.0 |
| 329 | 1.75 |
| 330 | 5.0 |
| 331 | 6.25 |
| 332 | 3.0 |
| 333 | 3.5 |
| 334 | 2.5 |
| 335 | 3.25 |
| 336 | 55.0 |
| 337 | 3.75 |
| 338 | 3.25 |
| 339 | 3.25 |
| 340 | 1.75 |
| 341 | 2.5 |
| 342 | 2.5 |
| 343 | 3.75 |
| 344 | 4.0 |
| 345 | 3.5 |
| 346 | 8.25 |
| 347 | 4.0 |
| 348 | 6.0 |
| 349 | 4.0 |
| 350 | 10.25 |
| 351 | 3.0 |
| 352 | 4.0 |
| 353 | 3.0 |
| 354 | 228.25 |
| 355 | 375.25 |
| 356 | 6.25 |
| 357 | 3.75 |
| 358 | 4.25 |
| 359 | 289.75 |
| 360 | 2.5 |
| 361 | 3.25 |
| 362 | 3.0 |
| 363 | 3.25 |
| 364 | 3.5 |
| 365 | 4.5 |
| 366 | 5.0 |
| 367 | 3.0 |
| 368 | 3.5 |
| 369 | 6.0 |
| 370 | 1.0 |
| 371 | 1.25 |
| 372 | 21.75 |
| 373 | 273.5 |
| 374 | 2.5 |
| 375 | 3.25 |
| 376 | 2.25 |
| 377 | 2.75 |
| 378 | 3.5 |
| 379 | 2.5 |
| 380 | 2.25 |
| 381 | 2.25 |
| 382 | 3.25 |
| 383 | 3.25 |
| 384 | 1.75 |
| 385 | 3.25 |
| 386 | 3.5 |
| 387 | 2.75 |
| 388 | 5.75 |
| 389 | 1072.25 |
| 390 | 1.25 |
| 391 | 2.25 |
| 392 | 1.75 |
| 393 | 3.25 |
| 394 | 3.0 |
| 395 | 2.0 |
| 396 | 4.25 |
| 397 | 11.5 |
| 398 | 3.25 |
| 399 | 4.25 |
| 400 | 2.5 |
| 401 | 2.25 |
| 402 | 2.5 |
| 403 | 1.5 |
| 404 | 8.0 |
| 405 | 5.25 |
| 406 | 1.5 |
| 407 | 2.0 |
| 408 | 1.75 |
| 409 | 10.5 |
| 410 | 3.0 |
| 411 | 5.0 |
| 412 | 8.5 |
| 413 | 16.5 |
| 414 | 3.25 |
| 415 | 5.5 |
| 416 | 0.75 |
| 417 | 3.0 |
| 418 | 2.5 |
| 419 | 410.75 |
| 420 | 4.75 |
| 421 | 7.5 |
| 422 | 3.0 |
| 423 | 2.25 |
| 424 | 2.0 |
| 425 | 2.75 |
| 426 | 1.5 |
| 427 | 2.25 |
| 428 | 1.75 |
| 429 | 2.75 |
| 430 | 2.5 |
| 431 | 3.0 |
| 432 | 2.25 |
| 433 | 9.0 |
| 434 | 3.5 |
| 435 | 2.0 |
| 436 | 4.0 |
| 437 | 4.75 |
| 438 | 206.75 |
| 439 | 10.75 |
| 440 | 4.0 |
| 441 | 2.5 |
| 442 | 2.0 |
| 443 | 2.5 |
| 444 | 1.25 |
| 445 | 7.75 |
| 446 | 10.0 |
| 447 | 2.75 |
| 448 | 4.75 |
| 449 | 3.5 |
| 450 | 2.0 |
| 451 | 3.5 |
| 452 | 2.75 |
| 453 | 1.75 |
| 454 | 2.25 |
| 455 | 1.75 |
| 456 | 1.25 |
| 457 | 3.0 |
| 458 | 2.5 |
| 459 | 1.25 |
| 460 | 2.5 |
| 461 | 2.5 |
| 462 | 3.25 |
| 463 | 5.25 |
| 464 | 2.75 |
| 465 | 9.5 |
| 466 | 5.0 |
| 467 | 4.5 |
| 468 | 6.25 |
| 469 | 1.0 |
| 470 | 17.0 |
| 471 | 2.5 |
| 472 | 3.5 |
| 473 | 1.25 |
| 474 | 3.75 |
| 475 | 3.25 |
| 476 | 5.0 |
| 477 | 11.0 |
| 478 | 3.75 |
| 479 | 3.0 |
| 480 | 2.0 |
| 481 | 3.0 |
| 482 | 3.25 |
| 483 | 2.25 |
| 484 | 5.0 |
| 485 | 12.75 |
| 486 | 4.0 |
| 487 | 1.75 |
| 488 | 6.75 |
| 489 | 3.25 |
| 490 | 1.75 |
| 491 | 3.75 |
| 492 | 5.5 |
| 493 | 9.75 |
| 494 | 3.75 |
| 495 | 2.5 |
| 496 | 2.5 |
| 497 | 4.5 |
| 498 | 6.0 |
| 499 | 1.75 |
| 500 | 1.5 |
| 501 | 2.5 |
| 502 | 2.25 |
| 503 | 3.0 |
| 504 | 2.5 |
| 505 | 1.5 |
| 506 | 2.75 |
| 507 | 2.75 |
| 508 | 18.75 |
| 509 | 2.75 |
| 510 | 1.75 |
| 511 | 1.75 |
| 512 | 2.25 |
| 513 | 1.75 |
| 514 | 1.75 |
| 515 | 1.75 |
| 516 | 2.25 |
| 517 | 2.5 |
| 518 | 1.25 |
| 519 | 1.0 |
| 520 | 2.25 |
| 521 | 1.25 |
| 522 | 2.25 |
| 523 | 2.5 |
| 524 | 2.75 |
| 525 | 9.5 |
| 526 | 428.0 |
| 527 | 2.25 |
| 528 | 1.75 |
| 529 | 1.5 |
| 530 | 8.5 |
| 531 | 18.25 |
| 532 | 1.75 |
| 533 | 1.5 |
| 534 | 4.75 |
| 535 | 22.0 |
| 536 | 2.5 |
| 537 | 8.75 |
| 538 | 4.0 |
| 539 | 1.5 |
| 540 | 1.25 |
| 541 | 3.5 |
| 542 | 0.5 |
| 543 | 3.0 |
| 544 | 1.0 |
| 545 | 10.75 |
| 546 | 16.5 |
| 547 | 8.75 |
| 548 | 2.0 |
| 549 | 2.0 |
| 550 | 2.25 |
| 551 | 6.5 |
| 552 | 9.0 |
| 553 | 3.0 |
| 554 | 5.25 |
| 555 | 2.0 |
| 556 | 2.25 |
| 557 | 7.5 |
| 558 | 11.75 |
| 559 | 2.25 |
| 560 | 4.75 |
| 561 | 9.25 |
| 562 | 10.0 |
| 563 | 9.25 |
| 564 | 5.25 |
| 565 | 5.75 |
| 566 | 24.5 |
| 567 | 1.5 |
| 568 | 1.5 |
| 569 | 2.0 |
| 570 | 16.0 |
| 571 | 2.75 |
| 572 | 2.0 |
| 573 | 1.5 |
| 574 | 1.5 |
| 575 | 1.75 |
| 576 | 3.0 |
| 577 | 2.5 |
| 578 | 4.25 |
| 579 | 2.0 |
| 580 | 1.75 |
| 581 | 14.5 |
| 582 | 13.25 |
| 583 | 4.25 |
| 584 | 11.75 |
| 585 | 2.25 |[389]
[249]
[526]
Figure S4
[389]
[249]
[526]
