# Supplementary material for: Fucosidases from the human gut symbiont Ruminococcus gnavus
Source: Cell Mol Life Sci. 2020 Apr 24;78(2):675–93. doi: 10.1007/s00018-020-03514-x (PMC7872956; doi:10.1007/s00018-020-03514-x)
Supplement: Supplementary file 2 — Supplementary file2 (DOCX 30 kb) [file 18_2020_3514_MOESM2_ESM.docx]

Supplementary Table S1: Primers used in this study.

| **Enzyme** | **Forward primer (5’-3’)** | **Reverse primer (5’-3’)** |
| --- | --- | --- |
| E1_10125 | AAGTTCTGTTTCAGGGCCCGGAAACAACAAATTGGATTGG | ATGGTCTAGAAAGCTTTAACCATTGATATACTGCGGCTG |
| E1_10180 | AAGTTCTGTTTCAGGGCCCGATGGAACCAAAAAAAGCACC | ATGGTCTAGAAAGCTTTACTCCTTTCCCGTAAAATGG |
| ATCC_03833 | CATAGCATATGAATCAGGAAATATGGAATC | CATAGCTCGAGCTATTTTTTCAACTCGATTTTC |
| ATCC_00842 | CATAGGGATCC ATGGAAGAATCTCAGGACAGG | CATAGCTCGAGTTAATTATCAATCTTTCCTGCAG |
| E1_10125G260M | GGTATATGCTTCTGCCATAAATAACATACAGTCTGCATCATATCCCG | CGGGATATGATGCAGACTGTATGTTATTTATGGCAGAAGCATATACC |

Supplementary Table S2: *In silico* analysis of putative *R. gnavus* GH29 and GH95 fucosidase protein sequences

| GH family | Strain | Protein name (abbreviation) | Size (aa) | MW (kDa) | pI | Signal peptide^1^ | Transmembrane helix^2^ | Presence of LPxTG motif^2^ | Predicted subcellular localization |
| --- | --- | --- | --- | --- | --- | --- | --- | --- | --- |
| GH29 | E1 | RUGNEv3_10125 (E1_10125) | 1312 | 144.3 | 4.91 | 1-33 | In 1-11; TM 12-34; Out 35-1312 | No | Unknown |
| GH29 | E1 | RUGNEv3_10180 (E1_10180) | 453 | 52.6 | 5.01 | No | No | No | Cytoplasmic |
| GH29 | E1 | RUGNEv3_10623 (E1_10623) | 478 | 54.9 | 4.85 | No | No | No | Unknown |
| GH29 | E1 | RUGNEv3_11127 (E1_11127) | 435 | 50.5 | 5.24 | No | No | No | Cytoplasmic |
| GH29 | ATCC 29149 | RUMGNA_03411 (ATCC_03411) | 1773 | 192.3 | 4.67 | 1-40 | In 1-20; TM 21-38; Out 39-1749; TM 1750-1769; In 1770-1773 | Out 1-1750; TM 1751-1769; In 1770-1773 | Cell wall |
| GH29 | ATCC 29149 | RUMGNA_03833 (ATCC_03833) | 435 | 50.5 | 5.14 | No | No | No | Cytoplasmic |
| GH95 | E1 | RUGNEv3_10181 (E1_10181) | 747 | 85.4 | 5.45 | No | No | No | Cytoplasmic |
| GH95 | E1 | RUGNEv3_10587 (E1_10587) | 745 | 85.7 | 5.29 | No | No | No | Cytoplasmic |
| GH95 | E1 | RUGNEv3_30029 (E1_30029) | 775 | 89.2 | 5.83 | No | No | No | Cytoplasmic |
| GH95 | E1 | RUGNEv3_40027 (E1_40027) | 2181 | 239.1 | 4.57 | 1-35 | In 1-11; TM 12-34; Out 35-2153; TM 2154-2176; In 2177-2181 | Out 1-2155; TM 2156-2176; In 2177-2181 | Unknown (Cell wall and/or extracellular) |
| GH95 | ATCC 29149 | RUMGNA_00842 (ATCC_00842) | 2168 | 237.3 | 4.64 | 1-35 | In 1-11; TM 12-34; Out 35-2140; TM 2141-2163; In 2164-2168 | Out 1-2142; TM 2143-2163; In 2164-2168 | Unknown (Cell wall and/or extracellular) |
| GH95 | ATCC 29149 | RUMGNA_01058 (ATCC_01058) | 784 | 90.2 | 5.83 | No | No | No | Cytoplasmic |
| GH95 | ATCC 29149 | RUMGNA_03121 (ATCC_03121) | 1873 | 211.6 | 4.70 | No | No | No | Unknown |

^1^ numbers are the amino acid positions of the predicted signal peptides; they are all predicted to be “standard” secretory signal peptides transported by the Sec translocon and cleaved by Signal Peptidase I

^2^ numbers are the amino acid positions of the different domains. In, inside/periplasmic space; TM, transmembrane domain; Out, outside/extracellular space

Supplementary Table S3: LC-MS/MS analysis for *R. gnavus* recombinant fucosidases against various fucosylated glycans

| **Substrate** | **Fucosidase** | | | |
| --- | --- | --- | --- | --- |
|  | **E1_10125**  (GH29) | **E1_10180**  (GH29) | **ATCC_03833**  (GH29) | **ATCC_00842**  (GH95) |
| sLeA | α-1,3-fucosidase | N.T. | α-1,4-fucosidase | N.D. |
| sLeX | α-1,4-fucosidase | N.T. | N.D. | N.D. |
| Sialylated human plasma N-glycans | α-1,3/4-fucosidase | N.T. | α-1,6-fucosidase | N.D. |
| De-sialylated human plasma N-glycans | α-1,3/4-fucosidase | N.T. | α-1,6-fucosidase | N.D. |
| Horseradish peroxidase N-glycans | N.D. | N.T. | N.D. | N.T. |
| Blood group A type II | N.D. | N.T. | N.T. | N.T. |
| Blood group B type II | N.D. | N.T. | N.T. | N.T. |
| FA2G2 | N.T. | N.T. | α-1,6-fucosidase | α-1,6-fucosidase |

N.D., not determined. N.T., not detected.

Supplementary Table S4: Specific activity of *R. gnavus* GH 29 fucosidase E1_10125 and E1_10125G260M against LeX and sLeX

|  | **E1_10125** | **E1_10125** | **E1_10125G260M** | **E1_10125G260M** |
| --- | --- | --- | --- | --- |
|  | U/µmol | V/E in s-1 | U/µmol | V/E in s-1 |
| LeX | 101.56 ± 3.79 | 1.69 ± 0.06 | 77.33 ± 6.29 | 1.29 ± 0.10 |
| sLeX | 53.61 ± 8.70 | 0.89 ± 0.14 | 15.15 ± 2.62 | 0.25 ± 0.04 |

Each enzyme (0.01 µM) was incubated with the substrate (0.1 mM) at 37°C in 50 mM citrate buffer at pH 6 for 15 min. One unit of enzyme activity was defined as the amount of enzyme (µmol) that releases 1 µmol Fuc per minute under experimental conditions.

Supplementary Table S5: Thermodynamics parameters of *R. gnavus* GH29 E1_10125 fucosidase binding to fucosylated ligands

|  | **LeX** | **sLeX** | **αGal-LeX** | **Neu5Ac** | **l-Fucose** |
| --- | --- | --- | --- | --- | --- |
| *K*_d_ (µM) | 51.4 ± 1.93 | 3.59 ± 0.48 | 47.1 ± 5.59 | 1.80 10^4^ ± 3.24 10^3^ | 21.7 ± 2.77 |
| ΔH (kcal/mol) | -4.62 ± 0.31 | -6.69 ± 0.04 | -5.17 ± 0.32 | -52.2 ± 14.4 | -8.40 ± 0.95 |
| ΔS (kcal/K/mol) | 4.15 10^-3^ ± 1.11 10^-3^ | 2.49 10^-3^ ± 2.77 10^-4^ | 1.75 10^-3^ ± 3.34 10^-4^ | -0.16 ± 0.04 | -8.82 10^-3^ ± 1.90 10^-4^ |
| ΔG (kcal/mol) | -5.85 ± 0.02 | -7.43 ± 0.08 | -5.91 ± 0.07 | -2.39 ± 0.10 | -6.37 ± 0.08 |
| N (sites) | 1.03 ± 0.04 | 0.90 ± 0.04 | 1.06 ± 0.09 | 1^*^ | 0.83 ± 0.04 |

The experiments were performed using the PEAQ-ITC system. The cell protein concentration was 100 µM and the syringe ligand concentration was 2 mM for all ligands tested except 20 mM for Neu5Ac.

^*^N was manually set to 1.
